# The role of the serotonin receptor subtypes 5-HT_1A_ and 5-HT_7_ and its interaction in emotional learning and memory

**DOI:** 10.3389/fphar.2015.00162

**Published:** 2015-08-07

**Authors:** Oliver Stiedl, Elpiniki Pappa, Åsa Konradsson-Geuken, Sven Ove Ögren

**Affiliations:** ^1^Department of Functional Genomics, Behavioral Neuroscience Group, Center for Neurogenomics and Cognitive Research, Neuroscience Campus Amsterdam – VU University AmsterdamAmsterdam, Netherlands; ^2^Department of Molecular and Cellular Neurobiology, Behavioral Neuroscience Group, Center for Neurogenomics and Cognitive Research, Neuroscience Campus Amsterdam –VU University AmsterdamAmsterdam, Netherlands; ^3^Department of Neuroscience, Karolinska InstitutetStockholm, Sweden

**Keywords:** emotional learning, fear conditioning, fear memory, 5-HT_1A_ receptor ligands, 5-HT_7_ receptor ligands, passive avoidance, serotonin

## Abstract

Serotonin [5-hydroxytryptamine (5-HT)] is a multifunctional neurotransmitter innervating cortical and limbic areas involved in cognition and emotional regulation. Dysregulation of serotonergic transmission is associated with emotional and cognitive deficits in psychiatric patients and animal models. Drugs targeting the 5-HT system are widely used to treat mood disorders and anxiety-like behaviors. Among the fourteen 5-HT receptor (5-HTR) subtypes, the 5-HT_1A_R and 5-HT_7_R are associated with the development of anxiety, depression and cognitive function linked to mechanisms of emotional learning and memory. In rodents fear conditioning and passive avoidance (PA) are associative learning paradigms to study emotional memory. This review assesses the role of 5-HT_1A_R and 5-HT_7_R as well as their interplay at the molecular, neurochemical and behavioral level. Activation of postsynaptic 5-HT_1A_Rs impairs emotional memory through attenuation of neuronal activity, whereas presynaptic 5-HT_1A_R activation reduces 5-HT release and exerts pro-cognitive effects on PA retention. Antagonism of the 5-HT_1A_R facilitates memory retention possibly via 5-HT_7_R activation and evidence is provided that 5HT_7_R can facilitate emotional memory upon reduced 5-HT_1A_R transmission. These findings highlight the differential role of these 5-HTRs in cognitive/emotional domains of behavior. Moreover, the results indicate that tonic and phasic 5-HT release can exert different and potentially opposing effects on emotional memory, depending on the states of 5-HT_1A_Rs and 5-HT_7_Rs and their interaction. Consequently, individual differences due to genetic and/or epigenetic mechanisms play an essential role for the responsiveness to drug treatment, e.g., by SSRIs which increase intrasynaptic 5-HT levels thereby activating multiple pre- and postsynaptic 5-HTR subtypes.

## Introduction

Serotonin (5-HT) is a biogenic amine acting as a neurotransmitter and neuromodulator. The distribution of serotonin-containing neurons in the CNS have been studied in different species and have been found to be localized exclusively in the brainstem (Hunt and Lovick, 1982; Takahashi et al., 1986; Ishimura et al., 1988). The majority of the serotonergic cell bodies reside in the dorsal and median raphe nuclei but send axons almost to the entire brain, including cortical, limbic, midbrain, and hindbrain regions (Charnay and Léger, 2010). As expected from the wide projection pattern of the 5-HT neurons, serotonin modulates variable physiological functions, such as sleep, arousal, feeding, temperature regulation, pain, emotions, and cognition (Bradley et al., 1986; Barnes and Sharp, 1999; Ögren et al., 2008; Berger et al., 2009; Artigas, 2015).

The pleiotropic behavioral effects of 5-HT are mediated by a family of at least 14 5-HTR subtypes (Hoyer et al., 1994). These 5-HTR subtypes are distributed in a brain- and cell-specific manner and regulate distinct physiological processes, through different and sometimes opposing signaling pathways (Hoyer and Martin, 1997; Hoyer et al., 2002).

The 5-HT_1A_R is one of the best-studied 5-HTR subtypes due to its implication in anxiety-like behaviors (Heisler et al., 1998; Parks et al., 1998; Toth, 2003), in depression (Lucki, 1991) as well as in cognitive processes that are impaired in several psychiatric disorders (review by Ögren et al., 2008; Millan et al., 2012). Its potential role as a drug target has been also investigated (Tunnicliff, 1991; Den Boer et al., 2000; Blier and Ward, 2003). The most common antidepressants, the SSRIs, act by targeting the 5-HT_1A_R (Hervas and Artigas, 1998; Artigas, 2015), supporting the key role of the 5-HT_1A_R in the pathophysiology of mood disorders.

The 5-HT_7_Rs are implicated in depression and anxiety, and evidence has been provided for their role in learning and memory (reviewed by Leopoldo et al., 2011). Interestingly, the 5-HT_7_R and 5-HT_1A_R exert opposing roles in the modulation of fear learning (Eriksson et al., 2008, 2012), pointing at the importance of both 5-HTR subtypes and their signaling interaction in the regulation of emotional learning.

After a brief introduction about the characteristics of 5-HT_1A_ and 5-HT_7_R (distribution, signaling, and ligands), this review will focus on the role of 5-HT_1A_R, 5-HT_7_R as well as its interplay in emotional learning processes. The interaction between the 5-HT_1A_R and 5-HT_7_R signaling will be discussed and results of studies using different available 5-HT_1A_R and 5-HT_7_R ligands on fear learning tasks are summarized. A considerable extent of this review will also be dedicated to describe the region-specific effects of 5-HT_1A_R and 5-HT_7_R, via local rather than systemic administration. Overall, the aim of this review is to draw general conclusions about the role of both 5-HT_1A_R and 5-HT_7_R in fear learning, which may contribute to our better understanding of the mechanisms underlying dysregulated learning and memory in affective disorders. The focus here is on fear learning because this one-trial learning task allows for exact timing of pharmacological manipulations to discriminate between different memory phases.

## Characteristics of the 5-HT_1A_ and 5-HT_7_ Receptors

All the 5-HTR subtypes belong to the G protein-coupled receptor superfamily, with the exception of the 5-HT_3_R as ionotropic receptor (Hoyer et al., 2002). The metabotropic 5-HTR subtypes consist of seven transmembrane domains and are classified into four groups based on the type of G proteins to which they are coupled. The 5-HT_1_Rs (5-HT_1A_R, 5-HT_1B_R, 5-HT_1D_R, 5-HT_1E_R, 5-HT_1F_R) couple to Gα_i_/Gα_o_ proteins, whereas the 5-HT_2_Rs (5-HT_2A_R, 5-HT_2B_R, 5-HT_2C_) couple to Gα_q_ proteins, and the 5-HT_4_R, 5-HT_6_R, and 5-HT_7_R couple to Gα_s_ proteins. For the 5-HT_5_Rs (5-HT_5A_R and 5-HT_5B_R) G-protein coupling is not established yet (Bockaert et al., 2006).

### 5-HT_1A_ Receptor Localization

5-HT_1A_R was the first 5-HTR subtype to be cloned and is characterized by its high affinity for 5-HT (Nichols and Nichols, 2008). 5-HT_1A_Rs are widely distributed throughout the CNS and are present in both pre- and postsynaptic sites. Presynaptically, 5-HT_1A_Rs are exclusively located on the cell bodies and dendrites of 5-HT neurons in the dorsal and median raphe nuclei (Riad et al., 2000) and function as 5-HT_1A_ autoreceptors which tightly regulate 5-HT neuronal activity.

Postsynaptically, the highest level of 5-HT_1A_R is found in the limbic system based on receptor autoradiography and mRNA expression. Both techniques showed the distribution of the 5-HT_1A_R in the lateral septum, cingulate and entorhinal cortices, with particularly high expression in the hippocampus (reviewed by Hannon and Hoyer, 2008). At the cellular level, the postsynaptic 5-HT_1A_R is expressed in cortical pyramidal neurons as well as pyramidal, GABAergic and granular cells of the hippocampus (Hannon and Hoyer, 2008). At least in the hippocampal formation, the 5-HT_1A_R is located on somata and dendrites of pyramidal and granular neurons, as well as on the dendritic spines of pyramidal neurons (Riad et al., 2000). Moreover, 5-HT_1A_R immunoreactivity has been demonstrated in different subgroups of neurons in the septal complex with GABAergic septohippocampal parvalbumin-containing projection neurons, GABAergic calbindin D-28-containing neurons as well as cholinergic septohippocampal neurons (Lüttgen et al., 2005a). This indicates that systemic administration of 5-HT_1A_R ligands can modify hippocampal function through effects on septohippocampal neurons that are responsible for the theta rhythm which plays an important role in memory functions (Elvander-Tottie et al., 2009).

### 5-HT_1A_ Receptor Signaling

Activation of 5-HT_1A_R leads to neuronal hyperpolarization, an effect mediated by pertussis-toxin-sensitive Gα_i/o_ proteins. Gα_i/o_ proteins are negatively coupled with the signaling pathway of adenylyl cyclase and thereby decrease the cAMP formation (De Vivo and Maayani, 1986; Weiss et al., 1986). Despite their high density in the dorsal raphe nucleus, 5-HT_1A_ autoreceptors do not seem to inhibit AC, but mediate neuronal inhibition through different signaling pathways (Clarke et al., 1996). Both post- and presynaptic 5-HT_1A_Rs inhibit neuronal firing via the activation of G protein-coupled inwardly rectifying potassium channels as well as the inhibition of Ca^2+^ channels (Sodickson and Bean, 1998; Bockaert et al., 2006). A multitude of other signaling pathways and effectors has been also linked to the activation of the 5-HT_1A_R (reviewed by Raymond et al., 2001; Bockaert et al., 2006).

### 5-HT_7_R Localization

The 5-HT_7_R was the last 5-HTR subtype to be cloned by using a targeted screening analysis of mammalian cDNA libraries and probes from already known receptors (Bard et al., 1993; Lovenberg et al., 1993; Ruat et al., 1993). Although 5-HT_7_Rs demonstrate a high interspecies homology (>90%; To et al., 1995), they share a low homology with the other 5-HTR subtypes (<50%; Bard et al., 1993). Northern blot analysis and *in situ* hybridization studies demonstrate high expression of 5-HT_7_R in the CNS and particularly in the hypothalamus (suprachiasmatic nucleus), thalamus, hippocampus, and cerebral cortex (Bard et al., 1993; Lovenberg et al., 1993; Ruat et al., 1993). Like 5-HT_1A_R, the 5-HT_7_R is also localized in the raphe nuclei in both rodent and human brain, which has raised questions about its role in the regulation of 5-HT levels (Martin-Cora and Pazos, 2004). At the neuronal level, 5-HT_7_R is expressed in hippocampal CA pyramidal neurons with a higher density in CA3 than in CA1 (Bonaventure et al., 2004) and a differential expression, with selective localization on the cell bodies in CA1 pyramidal neurons (Bickmeyer et al., 2002). Little is known, however, about the expression patterns of 5-HT_7_R in cortical neurons, where it is suggested that 5-HT_7_R may have a role during the developing stages of cortical circuits (Béïque et al., 2007; Celada et al., 2013).

### 5-HT_7_ Receptor Signaling

5-HT_7_R activation activates adenylyl cyclase signaling and consequently the conversion of ATP to cAMP through coupling to Gα_s_ (Bard et al., 1993; Lovenberg et al., 1993; Ruat et al., 1993). Although cAMP activation is commonly mediated by the PKA, it has been demonstrated that Epac, a member of the cAMP-regulated guanine nucleotide exchange family, has a crucial role in PKA-independent signaling (Lin et al., 2003). For instance, 5-HT_7_Rs activate the MAPK/ERK signaling pathway (Errico et al., 2001; Norum et al., 2003) via the stimulation of the Epac factor (Lin et al., 2003). Binding of cAMP to Epac leads to the activation of several other signaling pathways (reviewed by Holz et al., 2006).

## Functional Roles of 5-HT_1A_R and 5-HT_7_ Receptors

The expression of 5-HT_1A_R and 5-HT_7_R in the limbic system (Hannon and Hoyer, 2008; Berumen et al., 2012) support a role in the modulation of functions like mood, memory processing as well as emotional association with memory. The 5-HT_1A_R has been proposed to modulate anxiety based on studies with 5-HT_1A_R knockout mice (Heisler et al., 1998; Parks et al., 1998; Toth, 2003) and the response to antidepressant drugs (Blier and Ward, 2003; Artigas, 2015). Several partial 5-HT_1A_R agonists, e.g., buspirone, have been used to treat anxiety and depression (Tunnicliff, 1991; Den Boer et al., 2000), whereas co-administration of pindolol (β-adrenergic and 5-HT_1A_R antagonist) with SSRIs enhances their therapeutic efficacy and shortens their onset of action (reviewed by Artigas et al., 2001). A considerable body of literature demonstrates the 5-HT_1A_R involvement in various hippocampus-dependent learning and memory tasks (reviewed by Ögren et al., 2008).

In contrast, the available data on the function of 5-HT_7_R is relatively limited, mainly due to the lack of selective agonists specific for this 5-HTR subtype (Misane and Ögren, 2000; Nichols and Nichols, 2008; Leopoldo et al., 2011). The physiological role of 5-HT_7_R has been closely linked with the regulation of sleep, circadian rhythm, pain and also mood (reviewed by Leopoldo et al., 2011). Accumulating data implicates the 5-HT_7_R in the action of antidepressant drugs, whereas the results from anxiety studies are contradictory (Leopoldo et al., 2011). Interestingly, studies using 5-HT_7_R knockout mice revealed the crucial role of this receptor in hippocampus-dependent memory (Roberts et al., 2004; Sarkisyan and Hedlund, 2009).

## 5-HT_1A_ and 5-HT_7_ Receptor Ligands

### General Receptor Ligand Principles

Agents that act as receptor ligands may be agonists or antagonists. Agonists initiate physiological changes by activating downstream signaling pathways, whereas antagonists bind to receptors without producing any effect (Rang et al., 2015). Ligands can be divided in three categories based on their function:

(1) Full agonists produce a maximal response equivalent to the endogenous agonist (here 5-HT). These agonists have high efficacy (i.e., the ability to initiate changes which leads to effects) for the binding receptor.
(2) Partial agonists are not capable of producing the maximal functional response even when they occupy the entire receptor population. These agonists present intermediate efficacy. Respectively, we could refer to partial antagonists that bind to the active site (competitive antagonism) but do not completely abolish the receptor-mediated effects.
(3) Mixed profile ligands that (appear to) act both as agonists and as antagonist in distinct receptor populations. More likely, they have different agonist profiles at different receptor sites (e.g., pre-versus postsynaptic 5-HT_1A_R) and therefore appear to exert antagonist function in the presence of a full agonist, while acting as weak (partial) agonist thereby lowering the efficacy of the full agonist.

The function of any ligand used to study the role of 5-HT_1A_R and 5-HT_7_R is essential for the correct interpretation of the behavioral outcome. It is also important to mention that the intrinsic efficacy of a ligand is equally depended on the characteristics of response system; in our case the different brain populations of 5-HT_1A_R and 5-HT_7_R and their downstream signaling pathways. Agonists acting on the same receptor can produce different effects depending on their physicochemical properties, brain distribution, full or partial agonism as well as the number of coupled receptors in a brain area. The specificity of the compounds used is another very important characteristic that should be always taken into consideration and is referred to the ligand’s specific binding to the targeted receptor. Ligands with low specificity cannot be used to clarify the functional role of 5-HT_1A_R and 5-HT_7_R, since the produced effects can be also mediated via the binding to other proteins than the receptor of interest.

The physicochemical properties of compounds play an essential role for the drug uptake and diffusion with lipophilicity, solubility and molecular mass being among the most important properties (Waterhouse, 2003). The lipophilic nature of ligands is particularly important when they are administered locally. Increasing lipophilicity leads to enhanced blood–brain barrier diffusion, prevents the drug restriction in the area of interest and consequently produces wider effects, despite local application. This is evident from dorsohippocampal infusion of the blood–brain barrier penetrating drug 8-OH-DPAT, a full 5-HT_1A_R agonist, which impairs tone-dependent memory (Stiedl et al., 2000a), whereas this does not occur when the NMDAR antagonist APV (Stiedl et al., 2000b) and the GABA_A_R agonist muscimol are locally applied (Misane et al., 2013). The latter study is one of the few demonstrating the selective drug action in the dorsal hippocampus based on fluorescently labeled muscimol as bodipy conjugate. Besides the solubility of compounds and the applied dose, it is thus of high importance to consider other physico-chemical properties, such as half-life *in vivo*, to avoid misleading conclusions due to their wider spread (e.g., diffusion or potential active transport) in brain outside the target sites. The molecular weight of compounds can also provide valuable information about the diffusion capacity.

### 5-HT_1A_ Receptor Agonists

The prototypic 5-HT_1A_R agonist 8-OH-DPAT was the first full agonist developed (Arvidsson et al., 1981; Gozlan et al., 1983) and is still the most widely used to study the functional role of 5-HT_1A_R in behavioral manipulations (Barnes and Sharp, 1999). Despite its high selectivity for the 5-HT_1A_R, 8-OH-DPAT also acts as a 5-HT_7_R agonist (Bickmeyer et al., 2002; Eriksson et al., 2008) and observed effects can be the result of an interplay between the two receptor subtypes (see below).

Additionally, several full and partial agonists have been synthesized (see **Table 1**), but only a few of them have been used in fear learning studies, such as the buspirone and tandospirone. Buspirone belongs to the arylpiperazine (partial) agonists (Hjorth and Carlsson, 1982) and acts also as antagonist with high specificity for the dopamine D_2_ receptor (Witkin and Barrett, 1986). Tandospirone (SM-3997) is a 5-HT_1A_R partial agonist and was initially studied for its anxiolytic properties in rats and mice (Shimizu et al., 1987). Similar to buspirone, tandospirone also exhibits dopamine antagonist action with a potency that is considerably lower than the one for the 5-HT_1A_R (Shimizu et al., 1987). An overview of currently available 5-HT_1A_R agonists is provided in **Table 1**.

**Table 1:** Selected overview on available 5-HT_1A_ receptor agonists and ligands with mixed profile (reported function as presynaptic agonist and postsynaptic antagonist).

Function	Compound	Receptor Specificity	MW	Solvent	BBB penetr.	Behavior	Reference
Full/partial	Alnespirone (S-20499)	5-HT_1A_ >> D_2_ >> 5-HT_1B,2_ >> α,β >> D_1_ >> H_1_ (pre-synaptic)	479	W	n.a.	A	Griebel et al. (1992)
Partial	Buspirone	5-HT_1A_ = D_2_ >> α_1_,α_2_	385.5	W	n.a.	A, L	Hjorth and Carlsson (1982), Quartermain et al. (1993)
Full	F-13640	5-HT_1A_ >> n.a.	393.1	w	Yes	N	Deseure et al. (2002), Heusler et al. (2010); GtP
Partial	F-13714	5-HT _1A_ >> 5-HT _1B-F,2-7_	n.a.	w	n.a.	PPI	Assié et al. (2006)
Full	F-15599	5-HT_1A_ (post-synaptic) >> 5-HT_1B-F,2-7_	394.1	n.a.	Yes	FST	Maurel et al. (2007), Newman-Tancredi et al. (2009); GtP
Full	Flesinoxan	5-HT_1A_ >> α_1_ (antagonist) >> D_2_	415.5	W	Yes	A	Ahlenius et al. (1991), Hadrava et al. (1995)
Partial	Ipsapirone (TVX Q 7821)	5-HT_1A_ >> α_1_ (antagonist)	401.5	w	Yes	A	Traber et al. (1984)
Partial/full	LY-228729	5-HT_1A_ >> 5-HT_1B_	n.a.	w	n.a.	L, FST	Swanson and Catlow (1992)
n.a.	NDO-008	5-HT_1A_ >> n.a.	n.a.	w	n.a.	L	Misane et al. (1998)
Full	8-OH-DPAT	5-HT _1A_ >> 5-HT_7_ >> 5-HT_4_ >> D_2_	328.3	w	Yes	A, L	Arvidsson et al. (1981), Hadrava et al. (1995)
Full/Partial	Osemozotan (MKC-242)	5-HT_1A_ >> α_1_	379.8	w	n.a.	A	Matsuda et al. (1995), Sakaue et al. (2003)
Partial	PRX-00023	5-HT _1A_ >> 5-HT _1B_>α_1_>α_2_	n.a.	w	n.a.	A	Becker et al. (2006)
Full	Repinotan (BAY x 3702)	5-HT _1A_ >> 5-HT_7_ >> α_1_>α_2_>5-HT_4_	400.5	HC1	Yes	L	De Vry et al. (1998), Schwarz et al. (2005)
Partial	Tandospirone (SM-3997)	5-HT_1A_ >> D_2_	383.5	w	n.a.	A, L	Shimizu et al. (1987)

Mixed profile	S-15535	n.a.	432.5	w	Yes	A, L	Millan et al. (1993), Carli et al. (1999)
Mixed profile	MDL-73005		n.a.	w	n.a.	L	Hajós-Korcsok et al. (1999), Bertrand et al. (2001)

*A, anxiety; BBB, blood–brain barrier; D: FST; forced swim test; GtP, guide to pharmacology, see http://guidetopharmacology.org/; HCl, soluble in acidified aqueous solution; L, learning and memory tests; N, nociception; n.a., not available; penetr., penetrance; PPI, pre-pulse inhibition; W, soluble in water and/or saline.*

### 5-HT_1A_ Receptor Antagonists

WAY-100635 and NAD-299 are the most commonly used selective antagonists in the study of the 5-HT_1A_R. Both ligands have high potencies and penetrate easily into the brain (Fletcher et al., 1996; Johansson et al., 1997; Stenfors et al., 1998). However, NAD-299 was found to have higher selectivity for the 5-HT_1A_R than WAY-100635 (Fletcher et al., 1996; Johansson et al., 1997).

The last years novel compounds have been used to assess the role of 5-HT_1A_R in emotional learning, such as the potent and selective 5-HT_1A_R antagonists SRA-333 (lecozotan; Skirzewski et al., 2010), MC18 fumarate and VP08/34 fumarate (Siracusa et al., 2008; Pittalà et al., 2015).

The agents that were initially used as 5-HT_1A_R antagonist were 2-methoxyphenylpiperazine derivatives with structural similarity to buspirone, such as BMY-7378 and NAN-190 (Greuel and Glaser, 1992). However, these ligands were characterized as partial 5-HT_1A_R antagonist with antagonist properties only at the postsynaptic HT_1A_R and lower affinity for the α-adrenergic receptors (Greuel and Glaser, 1992).

Finally, S-15535 is reported to act as a postsynaptic 5-HT_1A_R antagonist while also behaving as an agonist on presynaptic 5-HT_1A_ autoreceptors, and therefore, it is characterized as a mixed profile ligand (Millan et al., 1993; Carli et al., 1999). However, a more recent study indicates predominantly weaker agonist activity of S-15535 at postsynaptic 5-HT_1A_Rs (Youn et al., 2009). An overview of currently available 5-HT_1A_R antagonists is provided in **Table 2**.

**Table 2:** Selected overview on available 5-HT_1A_ receptor antagonists.

Function	Compound	Receptor specificity	MW	Solvent	BBB penetr.	Behavior	Reference
Partial	BMY-7378	5-HT_1A_ >> α_1_ >> α*_2_* (partial agonist function) >> 5-HT_7_ >> 5-HT _1D_	385.9	W	+	A, L	Greuel and Glaser (1992), Grasby et al. (1992)
Partial	LY-426965	HT_1A_ >> 5-HT_1B_ (partial agonist function)	471.1	W	n.a.	A	Rasmussen et al. (2000); http://sis.nlm.nih.gov/
	MC18 fumarate	5-HT_1A_ >> n.a	515.7	W	n.a.	L	Pittalà et al. (2015)
	MP3022 NAD-299 (Robalzotan	5-HT_1A_ >> α_1_ >> 5-HT_2A_, α_2_, β, D_1_ and D_2_ 5-HT_1A_ >> α_1_,α_2_, β	351.5 354.9	n.a. W	n.a. +	n.a. A, L	Filip and Przegaliñski (1996) Johansson et al. (1997), Madjid et al. (2006); http://chem.sis.nlm.nih.gov/
Partial	NAN-190	HT_1A_ >> α_1_ (partial agonist function reported) >> 5-HT_R_, D	393.5	W	n.a.	A, L	Raghupathi et al. (1991), Greuel and Glaser (1992)
	p-MPPI	5-HT_1A_ >> α_1_	542.4	W	+	A	Kung et al. (1994), Allen et al. (1997); http://pubchem.ncbi.nlm.nih.gov/
	p-MPPF	5-HT_1A_ >> α_1_	507.4	n.a.	+	n.a.	Kung et al. (1996), Passchier et al. (2000); http://pubchem.ncbi.nlm.nih.gov/
	SB-649915	n.a., combined function as 5-HT_1A/B_ autoreceptor antagonist and SSRI	n.a.	MC	n.a.	A	Starr et al. (2007)
	Spiperone	5-HT_1A_ >> 5-HT_2A/c_ >> D_2_ antagonist and α_lb_ antagonist	n.a.	MC	n.a.	A	Starr et al. (2007)
	SRA-333 (Lecozotan)	5-HT_1A_ >> α_1_ >> D_2_ >> D_3_ >> D_4_ (α and D agonist)	n.a.	W	n.a.	A, L	Schechter et al. (2005)
	(S)-UH-301	5-HT_1A_ >> D_2_, D_3_ (agonist)	301.8	W	Yes	A, L	Moreau et al. (1992), Jackson et al. (1994)
	VP-08/34 fumarate	5-HT_1A_ >> n.a	513.6	W	Yes	L	Pittalà et al. (2015)
	WAY-100635	5-HT_1A_ >> α_1_ >> D_2_ >> D_3_ >> D_4_	538.6	W	Yes	A, L	Fletcher et al. (1996), Pike et al. (1996)
	WAY-405	5-HT_1A_ >> α	n.a.	MC	Yes	A, L	Minabe et al. (2003), Villalobos-Molina et al. (2005)
	WAY-101405	5-HT_1A_ >> n.a	n.a.	W	Yes	L	Hirst et al. (2008)

A, anxiety; BBB, blood–brain barrier; D: FST, forced swim test; L, learning and memory tests; MC, methylcellulose; n.a., not available; penetr., penetrance; PPI, pre-pulse inhibition; S*, 2-hydroxypropyl-β-cyclodextrin; W, soluble in water and/or saline.

### 5-HT_7_ Receptor Agonists

The lack of selective and potent 5-HT_7_R agonists (Misane and Ögren, 2000; Leopoldo, 2004; Leopoldo et al., 2011) is one of the major limitations to study the role of 5-HT_7_R in learning and memory. Currently, only a few selective 5-HT_7_R agonists exist and even less has been used in learning and memory studies. AS-19 and LP-44 are highly selective but low efficacy (partial) HT_7_R agonists whose functional role in fear learning was recently assessed (Eriksson et al., 2012). LP-211 is a novel highly selective 5-HT_7_R agonists (Leopoldo et al., 2008) but it has so far only been tested in an autoshaping Pavlovian/instrumental learning task (Meneses et al., 2015). An overview of currently available 5-HT_7_R agonists is provided in **Table 3**.

**Table 3:** Selected overview on available 5-HT_7_ receptor agonists and antagonists.

Function	Compound	Receptor specificity	MW	Solvent	BBB penetr.	Behavior	Reference
**Agonists**		
Partial	AS-19	5-HT_7_ >> n.a.	283.41	PG	n.a.	L, N	Brenchat et al. (2009), Eriksson et al. (2012)
Full	E-55888	n.a.	257.4	W	n.a.	N	Brenchat et al. (2009); http://pubchem.ncbi.nlm.nih.gov/
n.a.	LP-211	5-HT_7_ >> D_2_ > 5-HT_1A_	466.6	DMSO	Yes	L	Leopoldo et al. (2008), Meneses et al. (2015); http://pubchem.ncbi.nlm.nih.gov/
Partial	LP-44	5-HT_7_ >> 5-HT_1A_ (agonist function) >> 5-HT_2A_	488.1	PG	Yes	L, REM Sleep	Monti et al. (2008), Eriksson et al. (2012); http://pubchem.ncbi.nlm.nih.gov/
Partial	MSD-5a	5-HT_7_ >> 5-HT_1A_ >> 5-HT_2A_ >> D_2_	n.a.	W	n.a.	N	Thomson et al. (2004), Brenchat et al. (2009)
**Antagonists**		
	DR4004	5-HT_7_ >> 5-HT_2_ > D_2_ > HT_1A_ > HT_6_ > HT_4_	382.5	T80	A, L	n.a.	Kikuchi et al. (1999); http://pubchem.ncbi.nlm.nih.gov/
	SB-258719	5-HT_7_ >> 5-HT_1D_ >> D_2_, D_3_ >> 5- >> 5-HT_1B_,5-HT_2B_ >> HT_1A_	338.5	W	n.a.	N	Forbes et al. (1998), Brenchat et al. (2009); http://pubchem.ncbi.nlm.nih.gov/
	SB-269970^∗^	5-HT_7_ >> 5-HT_5A_ >> D_2_ > 5-HT_1B_ > HT_1D_	352.5	T80	Yes	A, FST, L	Lovell et al. (2000), Thomas et al. (2002), Wesolowska et al. (2006), Eriksson et al. (2012); http://pubchem.ncbi.nlm.nih.gov
	SB-656104-A	5-HT_7_ >> 5-HT_1D_ > 5-HT_2A_ >HT_2B_>D_2_ >5-HT_5A_	n.a	MC	Yes	L, REM Sleep	Thomas et al. (2003), Horisawa et al. (2011)
	SB-258741^∗∗^	5-HT_7_ >> 5-HT_1A_ > D_3_ > HT_1B_, D_2_ > 5-HT_1D_	350.5	W	n.a.	SZ	Lovell et al. (2000), Pouzet et al. (2002); http://pubchem.ncbi.nlm.nih.gov/

A, anxiety; BBB, blood–brain barrier; DMSO, dimethyl sulfoxide; FST, forced swim test; L, learning and memory tests; MC, methylcellulose; n.a., not available; penetr., penetrance; PG, propylene glycol; PPI, pre-pulse inhibition; SZ, schizophrenia assays; T80: Tween 80; W: soluble in water and/or saline; *behaves as quasi-full inverse agonist (Mahé et al., 2004); **behaves as partial inverse agonist (Mahé et al., 2004).

### 5-HT_7_ Receptor Antagonists

SB-258719 is the first selective 5-HT_7_R antagonist described (Forbes et al., 1998) but has not yet been used to investigate the role of 5-HT_7_R in the modulation of emotional learning. Both SB-656104-A and SB-269970 possess high potency and selectivity for 5-HT_7_R (Lovell et al., 2000; Thomas et al., 2002, 2003). These are the most commonly used 5-HT_7_R antagonists in behavior studies. An overview of currently available 5-HT_7_R antagonists is provided in **Table 3**.

## Behavioral Tasks for the Assessment of Emotional Learning and Memory

The experimental studies on emotional learning and memory in animals are based originally on psychological analysis of conflict behavior involving approach and avoidance of conditioned stimuli. Traditionally, the assays used to investigate animal behavior are based on the association of pleasant (i.e., motivationally related reward like food) or aversive stimuli (i.e., conditions related to negative feelings like pain and danger) to environmental cues involving classical (Pavlovian) or instrumental conditioning (Ögren and Stiedl, 2015).

The FC and the PA tasks are the most commonly used associative learning paradigms based on contextual fear learning. This type of learning is dependent on the operation of neuronal circuits in the limbic system, such as hippocampus and amygdala (Cahill and McGaugh, 1998; LeDoux, 2000) as demonstrated by us in mice (e.g., Stiedl et al., 2000a,b; Baarendse et al., 2008). Unlike FC, PA also includes instrumental learning. In the step-through PA test, the animal needs to suppress its innate preference for the dark compartment (where it previously received a foot shock) and remain in the bright compartment. In the step-down PA paradigm, however, the retention is examined in the dark compartment, where the animal received the foot shock (unconditioned stimulus) after stepping down from an elevated platform. The PA test procedure can be modified to examine any facilitating effect of the treatment on PA retention (Madjid et al., 2006). More specific information on the PA task is provided elsewhere (Ögren and Stiedl, 2015). A refined version of this task may provide for better translational aspects to assess pathological fear states such as post-traumatic-like responses based on deliberate choice of mice (Hager et al., 2014).

The single-trial learning design of FC and PA, which is sufficient to establish long-term and remote memory, allows the exact timing of the drug treatment in relation to training and retention test. Thereby, unlike multi-session tasks, one-trial tasks provide a unique advantage to study learning mechanisms as well as drug effects (here 5-HT_1A_R and 5-HT_7_R ligands) on the different phases of learning and memory, i.e., the acquisition phase that consists of encoding and early consolidation, consolidation, the recall (retrieval and expression) phase as well as the extinction phase and reconsolidation.

## Effects of 5-HT_1A_ Receptor Ligands in Emotional Learning and Memory

An overview of the behavioral effects of various 5-HT_1A_R ligands is provided in **Table 4**.

**Table 4:** Overview of the behavioral effects of 5-HT_1A_ receptor agonists, ligands with mixed profile and antagonists in fear learning tasks.

Compound	Species: Strain	Time of injection	Dose (mg/kg)	Admin. route	Behavior assay and behavioral consequences	Reference
**Agonists**		
Buspirone	M: Swiss-W.	30 min pretr.	1	s.c.	FC: reduced freezing in 24-h delay	Quartermain et al. (1993)
NDO-008	R: Sprague-D.	15 min pretr.	0.25–1.0	s.c.	PA: impaired PA retention at 24-h test	Misane et al. (1998)
8-OH-DPAT	M: C57BL/6J	15 min pretr.	0.05 and 1	s.c.	FC: impaired freezing at 1-h and 24-h test	Stiedl et al. (2000a)
		0 min post-tr.	0.05 and 1	s.c.	FC: no effect	Stiedl et al. (2000a)
		15 min pretr.	2 × 2.5 μg	i.h.	FC: impaired freezing at 24-h test	Stiedl et al. (2000a)
	M: C57BL/6J	15 min pretr.	0.3	s.c.	PA: impaired PA retention at 24-h test	Eriksson et al. (2012)
Tandospirone	M: Swiss-W.	30 min pretr.	2 and 5	s.c.	FC: reduced freezing at 24-h test	Quartermain et al. (1993)
	M: Swiss-W.	30 min pretr.	2 and 5	s.c.	FC: no effect at 1-h test	Quartermain et al. (1993)
	M: Swiss-W.	30 min pretest	2 and 5	s.c.	FC: no effect	Quartermain et al. (1993)
	M: Swiss W.	30 min pretr.	2.5 and 5	s.c.	PA: DD PA retention impairment	Mendelson et al. (1993)
**Mixed profile**		
MDL-73005	R: Long-E.	15 min pretr.	2	i.p	MWM: no effect alone but prevented the memory impairment induced by scopolamine (0.25 mg/kg)	Bertrand et al. (2001)
S15535	M: C57BL/6J	20 min pretr.	0.01–05	s.c.	FC: impairment at higher dose (>2 mg/kg)	Youn et al. (2009)
**Antagonists**		
BMY-7378	M: Swiss-W.	30 min pretr.	0–5	s.c.	PA: no effect	Mendelson et al. (1993)
MC18	M: C57BL/6J	15 min pretr.	0.1–1	s.c.	PA: U-shaped PA retention facilitation (maximum at 0.3 mg/kg)	Pittalà et al. (2015)
NAD-299	M: C57BL/6J	20 min pretr.	0.3 and 1	s.c.	FC: increased freezing at 24-h test	Youn et al. (2009)
	M: C57BL/6J	15 min pretr.	0.1–3	s.c.	PA: DD PA retention facilitation at 24-h test	Madjid et al. (2006)
	M: NMRI	15 min pretr.	0.1–3	s.c.	PA: U-shaped PA retention facilitation (maximum at 1 mg/kg)	Madjid et al. (2006)
SRA-333	R: Sprague-D.	30 min pretr.	0.3–2	s.c.	PA: DD PA retention facilitation	Skirzewski et al. (2010)
(S)-UH-301	R: Sprague-D.	30 min pretr.	0–3	s.c.	PA: no effect	Jackson et al. (1994)
VP-08/34	M: C57BL/6J	15 min pretr.	0.3 and 1	s.c.	PA: no effect	Pittalà et al. (2015)
WAY-100635	R: Sprague-D.	30 min pretr.	0.003–0.3	s.c.	PA: attenuated the PA retention deficit by PC A (0.03–0.1 mg/kg)	Misane and Ögren (2000)
	R: Wistar	30 min pretr.	1	i.p.	PA: reversed MK-801-induced memory impairment	Horisawa et al. (2011)
	R: Wistar	0 min post-tr.	0.01	i.v.	PA: reversed MK-801-induced memory impairment	Horisawa et al. (2011)
	R: Sprague-D.	120 min pretr.	3	po.	FC: Reversed scopolamine-induced memory deficits	Hirst et al. (2008)

*A, anxiety tests; DD, dose-dependent, FC, fear conditioning; i.h., intrahippocampal; i.p., intraperitoneal, i.v., intravenous; M, mice; n.a., not available; PA, passive avoidance; post-tr., post-training; p.o., per os; pretr, before training; R, rats; s.c., subcutaneous.*

### Systemic 5-HT_1A_ Receptor Ligand Effects

Despite the differences among the 5-HT_1A_R ligands in their chemical and pharmacological features (e.g., receptor selectivity and partial or full agonist properties; see **Tables 1** and **2**), there is strong evidence for the impairing effect of postsynaptic 5-HT_1A_R activation on fear memory. Systemic, pretraining administration of the full 5-HT_1A_R agonist 8-OH-DPAT shows a biphasic effect on PA performance, with the low dose range (0.01, 0.03 mg/kg) facilitating and the high dose range (0.1–1 mg/kg) impairing PA retention 24 h after training in both rats (Misane and Ögren, 2000; Lüttgen et al., 2005b) and mice (Madjid et al., 2006). The impairing dose of 8-OH-DPAT (0.2 and 0.3 mg/kg) also induces signs of the serotonin syndrome (Carli et al., 1992; Lüttgen et al., 2005b) linking the postsynaptic 5-HT_1A_R to the learning deficits. In line with these results, FC studies demonstrated that pretraining systemic injections of high doses (0.1–0.5 mg/kg) of 8-OH-DPAT impair fear learning (Stiedl et al., 2000a; Youn et al., 2009). Pretreatment with the selective 5-HT_1A_R antagonist WAY-100635 (0.03–1 mg/kg) blocked the impairment in freezing (FC) and transfer latency (PA), confirming and extending the detrimental role of the postsynaptic 5-HT_1A_R activation on memory acquisition.

The observed memory deficit was already present in short-term memory tests performed 1 h after training for FC retention (Stiedl et al., 2000a) and 5 min after PA training (Misane and Ögren, 2000). Thus, postsynaptic 5-HT_1A_R activation specifically impairs memory encoding of the aversive experience and not memory consolidation. In agreement to that observation, immediate 8-OH-DPAT post-training administration did not alter PA or FC retention (Misane and Ögren, 2000; Madjid et al., 2006).

### Local 5-HT_1A_ Receptor Ligand Effects

Intracranial administration of 5-HT_1A_R agonists and/or antagonists was used to further elucidate the distinct function of pre- versus postsynaptic 5-HT_1A_Rs in fear learning. Pre- but not post-training intra-hippocampal infusion of 8-OH-DPAT impairs contextual FC (Stiedl et al., 2000a), pointing at the important role of the postsynaptic 5-HT_1A_R in acquisition processes as observed after systemic administration.

## Effects of 5-HT_1A_ Receptor Agonists and Antagonists on Memory Recall

### Systemic 5-HT_1A_ Receptor Ligand Effects

Unlike the unambiguous implication of the postsynaptic 5-HT_1A_R in memory acquisition, its role in fear retrieval and expression is less clear. The systemic 5-HT_1A_R agonist NDO-008 (0.5 mg/kg) administered before the retention test to rats impairs slightly PA performance (Misane et al., 1998). In contrast, systemic administration of buspirone at the dose of 1 and 3 mg/kg had no effect on fear expression in mice (Quartermain et al., 1993). These different effects may partly depend on the readouts and the side effects elicited by higher 5-HT_1A_R dosages, such as the hypolocomotion induced together with the serotonin syndrome (Stiedl et al., 2000a). The hypolocomotion confounds the interpretation of fear expression results in mice when based on freezing. Moreover, it also possible that differences exists between rats and mice, although our own data shows high similarity of results in these two species.

Therefore, a recent study tried to clarify the role of the 5-HT_1A_R in fear recall, by assessing the effect of 8-OH-DPAT on fear-conditioned HR responses (reviewed by Stiedl et al., 2009) upon training and 24 h after training, in mice (Youn et al., 2013). Systemic pretest administration reduced the conditioned maximum HR as a consequence of the significantly reduced baseline HR before the presentation of the conditioned stimulus (tone). However, the tone-induced HR increase was preserved during the retention of auditory fear in mice with similar magnitude as compared to that in controls. Additionally, 8-OH-DPAT reduced the unconditioned tachycardia elicited by novelty exposure as a consequence of altered HR dynamics indicating autonomic dysregulation with enhanced parasympathetic function through postsynaptic 5-HT_1A_R activation (Youn et al., 2013). Thus, the claims of anxiolytic actions of pretest injection of 5-HT_1A_R agonists as initially reported in human studies and partly in animal models cannot be supported unambiguously at least in learned fear experiments.

### Local 5-HT_1A_ Receptor Ligand Effects

Local administration approaches tried to distinguish the role of the post- versus the presynaptic 5-HT_1A_R in the different aspects of fear expression. Bilateral microinjections of a selective 5-HT_1A_R agonist flesinoxan decreased the expression of conditioned contextual freezing when injected into the hippocampus or amygdala but not in the medial prefrontal cortex (Li et al., 2006), as well as the fear-potentiated startle responses when infused into the central amygdala (Groenink et al., 2000).

The role of 5-HT_1A_ autoreceptors in fear expression was also studied by pretest infusion of 8-OH-DPAT into the median raphe nuclei. This resulted in impaired contextual freezing responses (Borelli et al., 2005; Almada et al., 2009), but not fear-potentiated startle (Groenink et al., 2000; Almada et al., 2009) suggesting the existence of raphe-dependent serotonergic regulation that appears to modulate the freezing response to the aversive context. In contrast, hippocampal 8-OH-DPAT impaired the expression of both contextual freezing and fear-potentiated startle (Almada et al., 2009). However, 8-OH-DPAT mediates hyperlocomotion in rats (but hypolocomotion in mice) leading to a similar problem of potentially confounded interpretation of freezing performance during the drug state as mentioned before for mice.

## Effects of 5-HT_1A_ Receptor Agonists and Antagonists on Memory Extinction

In contrast to the well-studied implication of 5-HT_1A_Rs on memory acquisition and recall, there is only one study with 5-HT_1A_R ligands on fear extinction. The systemic 5-HT_1A_R agonist buspirone abolishes the fear extinction in mice (Quartermain et al., 1993). Similarly, the systemic 5-HT_1A_R antagonist WAY-100635 before a second sampling trial impaired the extinction of object recognition memory in rats (Pitsikas et al., 2003). Further studies are needed to determine the precise role of 5-HT_1A_Rs in memory extinction and/or reconsolidation in emotional learning tasks. Furthermore, local rather than systemic approaches are necessary to identify the neurocircuitry involved in these processes. The roles of other 5-HTRs in fear learning and the consequences of altered 5-HT neurotransmission on fear extinction are reviewed by Homberg (2012).

## Effects of 5-HT_7_ Receptor Agonists and Antagonists on Emotional Learning

### Systemic 5-HT_7_ Receptor Ligand Effects

The paucity of studies 5-HT_7_R functions on emotional learning is mainly due to the lack of selective ligands, especially agonists (Misane and Ögren, 2000; Leopoldo, 2004; Leopoldo et al., 2011; see **Table 5** and text above). Recent data from an autoshaping task showing that the 5-HT_7_R agonist, LP-211, when administered systematically after the training session, reversed scolopamine-induced amnesia, in rats (Meneses et al., 2015). The same group also shows a facilitating effect on memory formation by the 5-HT_7_R agonist AS-19 administered after an autoshaping training session (Perez-García and Meneses, 2005). The enhancing effect of 5-HT_7_Rs on memory consolidation was blocked by pre-injection of the 5-HT_7_R antagonist SB-269970 (Perez-García and Meneses, 2005; Meneses et al., 2015) indicating the specific involvement of the 5-HT_7_R.

**Table 5:** Overview of the behavioral effects of 5-HT_7_ receptor agonists and antagonists in learning tasks (not restricted to fear learning).

Compound	Species: Strain	Time of injection	Dose (mg/kg)	Admin. route	Behavior assay and behavioral consequences	References
**Agonists**		
AS-19	M: C57BL/6J	15 min pretr.	3–10	i.p.	DD activity reduction PA: no effect in retention latencies, 24 h after training	Eriksson et al. (2012)
	R: Wistar	0 min post-tr.	0.5–10.0	s.c	P/I-A: Enhanced memory consolidation, 24 h after training	Perez-García and Meneses (2005)
LP-211	R: Wistar	0 min post-tr.	0.1–10.0	i.p.	P/I-A: only 0.5 mg/kg had a possitive effect on memory consolidation, when tested 24 h after training	Meneses et al. (2015)
LP-44	M: C57BL/6J	15 min pretr.	1–10	i.p.	PA: DD activity reduction but no effect on PA retention latencies tested 24 h after training	Eriksson et al. (2012)
NAD-299 + 8-OH-DPAT	M: C57BL/6J	30 min +15 min pretr.	0.3 + 1	s.c.	PA: facilitates retention latencies 24 h after training serving as 5-HT_7_R activation	Eriksson et al. (2012)
**Antagonists**		
DR4004	R: Wistar	0 min post-tr.	0.5–10.	i.p.	P/I-A: no effect	Meneses (2004)
SB-269970	R: Wistar	0 min post-tr.	1–20	i.p.	P/I-A: no effect	Meneses (2004)
	M: C57BL/6J	30 min pretr.	20	s.c.	PA: reversed the facilitation by 8-OH-DPAT + NAD-299	Eriksson et al. (2012)
SB-656104-A	R: Wistar	60 min pretr.	10 and 30	i.p.	PA: reversed MK-801-induced memory impairment	Horisawa et al. (2011)
	R: Wistar	60 min pretr.	0.3	i.p.	PA: Counteracted the effect of MK-801	Horisawa et al. (2011)


*A, anxiety tests; DD, dose dependent, FC, fear conditioning; i.h., intrahippocampal; i.p., intraperitoneal, i.v., intravenous; M, mice, MSRAP, multiple schedule repeated acquisition performance; MWM, Morris water maze; n.a., not available; OR, object recognition task; OT, operant task; PA, passive avoidance; P/I-A, Pavlovian/instrumental autoshaping task; post-tr., post-training; p.o., per os; pretr, before training; R, rats; s.c., subcutaneous.*

Eriksson et al. (2008) investigated the role of 5-HT_7_R on emotional learning in mice using a step-through PA paradigm. Pretraining systemic administration of the 5-HT_7_R antagonist SB-269970 enhanced the impairing effect of low doses of 8-OH-DPAT (Eriksson et al., 2008). This result supports the notion that 5-HT_7_R activation has a beneficial modulatory role in learning opposing the function of 5-HT_1A_R activation. Accordingly, pretraining 5-HT_7_R activation by the combined use of the 5-HT_1A_R antagonist NAD-299 with the 5-HT_1A_R and 5-HT_7_R agonist 8-OH-DPAT facilitated PA retention (Eriksson et al., 2012). This PA facilitation by NAD-299 together with 8-OH-DPAT was again blocked by the 5-HT_7_R antagonist SB-269970 indicating a procognitive effect of 5-HT_7_R activation by this drug combination. However, the 5-HT_7_R agonists LP-44 and AS-19 failed to mediate this PA facilitation, despite dose-dependent tests. Despite their high *in vitro* potency to stimulate intracellular signaling cascades (Eriksson et al., 2012), the 5-HT_7_R agonists LP-44 and AS-19 have moderate agonist efficacy *in vivo*. This finding is in agreement with previous pharmacological characterization (Monti et al., 2008; Bosker et al., 2009; Brenchat et al., 2009) *in vivo* and may explain why the facilitatory effect of NAD-299 with 8-OH-DPAT could not be mimicked by the putative agonists LP-44 and AS-19.

### Local 5-HT_7_ Receptor Ligand Effects

To further address the role of 5-HT_7_Rs on emotional learning, Eriksson et al. (2012) performed hippocampal infusions with the 5-HT_7_R agonist AS-19 in mice. Since they failed to find clear facilitatory effects, as observed after systemic treatment, they concluded that “5-HT_7_Rs appear to facilitate memory processes in a broader cortico-limbic network and not the hippocampus alone.” The failure of the SB-269970 to enhance emotional memory, upon hippocampal infusions, may be the consequence of the low dose that can be locally infused due to the relatively poor solubility of SB-269970. However, systemic administration of this 5-HT_7_R antagonist fully blocked the PA facilitation observed after 5-HT_1A_R blockade. Hence, the hippocampus-dependent involvement of the 5-HT_7_Rs needs to be re-investigated with selective highly potent 5-HT_7_R agonists, because also the low potency of AS-19 (Eriksson et al., 2012) may have contributed to the lack of effects by dorsohippocampal 5-HT_7_R agonist application on PA. Finally, although the role of 5-HT_7_R in memory consolidation has been suggested, there are currently insufficient data supporting this view. More work is also required to clarify the role of 5-HT_7_R in memory extinction and reconsolidation, which are both essentially unexplored.

## The Interplay of the 5-HT_1A_ and 5-HT_7_ for Emotional Learning

The interaction of the two 5-HTR subtypes in emotional learning has been studied by using 8-OH-DPAT, which exerts agonistic effects for both 5-HT_1A_Rs and 5-HT_7_Rs. To dissect the function of these 5-HTRs, pre-treatment with selective 5-HT_1A_R antagonists is used to exclusively activate 5-HT_7_R. Eriksson et al. (2008) were the first to suggest the functional interplay between the two 5-HTRs on the behavioral level as the activation of 5-HT_7_R counteracted the 5-HT_1A_R-mediated impairments in PA performance. The interaction between the two 5-HTRs and their functional antagonism was then extended by experiments in mice, demonstrating that 5-HT_7_R activation and concomitant 5-HT_1A_R blockade leads to PA facilitation (Eriksson et al., 2012). The facilitatory effect on emotional memory by the 5-HT_1A_ antagonist NAD-299 was related to stimulation of 5-HT_7_Rs under conditions with reduced 5-HT_1A_R transmission. These findings suggest that the states of 5-HT_1A_Rs and 5-HT_7_Rs play a critical role for 5-HT effects on emotional memory. Consequently, the elevation of endogenous 5-HT via SSRIs will most likely result in differential cognitive/emotional effects depending on genetic and/or epigenetic regulation and occupancy of these two 5-HTRs in health and disease. This condition will affect the expression of the 5-HT_1A_R and change the relative balance between 5-HTR subtypes, which together will eventually determine the physiological actions of 5-HT and the clinical efficacy of SSRI treatment.

## Mechanisms Underlying the Functional Interaction of 5-HT_1A_R and 5-HT_7_R

As described above, 5-HT_1A_Rs and 5-HT_7_Rs mediate opposing effects regarding the neuronal excitability. 5-HT_1A_R activation reduces the activity of adenyl cyclase, whereas 5-HT_7_R activation stimulates adenyl cyclase activity and thereby increases intracellular cAMP thereby increasing neuronal excitability (Bockaert et al., 2006; Nichols and Nichols, 2008; Berumen et al., 2012). Accordingly, 5-HT_7_R stimulation in the hippocampus was found to activate pyramidal neurons, unlike 5-HT_1A_R activation which inhibited pyramidal neurons (Bickmeyer et al., 2002). Both 5-HTRs are expressed in glutamatergic hippocampal pyramidal neurons (Bockaert et al., 2006; Nichols and Nichols, 2008; Berumen et al., 2012). Therefore, it is likely that 5-HT_1A_R and 5-HT_7_R stimulation decreases and increases glutamate release in the hippocampus, respectively. In line with these results, 5-HT_7_R activation enhances the AMPA receptor-mediated synaptic currents on CA1 pyramidal neurons, whereas 5-HT_1A_R activation inhibits the AMPA receptor-mediated transmission between CA3 and CA1 pyramidal neurons in both pre- and postsynaptic sites (Costa et al., 2012). However, the 5-HT_1A_R-mediated inhibitory effect on glutamatergic neurotransmission was stronger than the 5-HT_7_R-mediated facilitatory effect (Costa et al., 2012). One explanation for the increased effectiveness of 5-HT_1A_R in controlling the input from the Schaffer collaterals may stem from the different localization of the two receptors on the CA1 pyramidal neurons: 5-HT_7_Rs are found on the cell bodies (Bickmeyer et al., 2002), whereas the 5-HT_1A_Rs appear to be mainly localized on dendrites (Kia et al., 1996).

Differences in the expression of the receptors could also play an essential role in their distinct activation pattern from the endogenous 5-HT. The progressive reduction of post-synaptic 5-HT_7_R levels during postnatal development, together with the maintenance of the expression level of 5-HT_1A_R (Kobe et al., 2012; Renner et al., 2012), could increase the ratio of membrane 5-HT_1A_Rs over 5-HT_7_Rs. Consequently, a model has been proposed regarding the molecular mechanisms that underlie the regulation of the 5-HT_1A_Rs and 5-HT_7_Rs. 5-HT_1A_R and 5-HT_7_R form heterodimers both *in vitro* and *in vivo* (Renner et al., 2012). This heterodimerization plays a functional role by decreasing G_i_ protein coupling of the 5-HT_1A_R and by reducing the ability of 5-HT_1A_R to activate potassium channels, without affecting the G_s_ protein coupling of the 5-HT_7_R. The heterodimerization additionally contributes to the desensitization of the 5-HT_1A_R through facilitated internalization (Renner et al., 2012).

5-HT_1A_R and 5-HT_7_R are co-localized in the cell membrane of hippocampal neurons, where their heterodimerization induces an inhibitory effect on the 5-HT_1A_R-mediated activation of potassium channels in hippocampal neurons (Renner et al., 2012). As mentioned above the post-synaptic levels of 5-HT_7_R are lower compared to the expression levels of post-synaptic 5-HT_1A_R, whereas this is not the case for the pre-synaptic 5-HT_7_R (Renner et al., 2012). These regional differences in the 5-HT_7_R levels and therefore in the concentration of the heterodimers, can explain the preferential desensitization of 5-HT_1A_ autoreceptors by SSRIs and more generally the region- and cell- specific differences in the signaling pathway mediated by the 5-HT_1A_R activation (see Naumenko et al., 2014). In summary, the above data suggest that the positive or negative consequences of a drug on emotional memory and cognition depend on the relative level of 5-HTR expression and, its efficacy in activating different receptors with their downstream signaling pathways.

## Genetic and Epigenetic Effects on 5-HT Transmission and Receptor Expression

Genetic and/or epigenetic effects regulate the receptor’s state and eventually define the physiological actions of endogenous 5-HT. A characteristic example is the Ala50Val variant of the 5-HT_1A_R, located in the transmembrane region 1, that leads to loss of response to 5-HT and consequently to the interruption of 5-HT signaling (Del Tredici et al., 2004). Moreover, the human polymorphism Gly22Ser attenuates the downregulating effect induced by long-term 8-OH-DPAT stimulation in comparison to the Val28 variant and wild-type without effect on the ligand binding capacity (Rotondo et al., 1997). It is suggested that individuals with the Ser22 variant have higher sensitivity to SSRIs treatment since its serotonergic effect depends on the efficiency of 5-HT_1A_R transmission (Rotondo et al., 1997). Furthermore, carriers of the short (s) allele of the 5-HT transporter promotor region possess behavioral abnormalities, such as increased levels of anxiety and FC as well as stronger fear potentiated startle (Bauer, 2014) in comparison to long (l) allele carriers. Accordingly, the therapeutic efficacy of SSRIs is reduced in patients homozygous for the s-allele when compared with heterozygous or l-allele carriers (Tomita et al., 2014).

The epigenetic regulation of 5-HTR subtypes is also implicated in the differential emotional and cognitive modulation induced by the serotonergic signaling. It is widely accepted that 5-HT_1A_R binding is reduced in the brain of depressed humans (e.g., Savitz et al., 2009) as well as in stressed rats (e.g., Choi et al., 2014) as indication of epigenetic modulation. 5-HT_1A_R activation in the basolateral amygdala and the prelimbic area of the prefrontal cortex in low-anxious rats reduced fear potentiated startle, whereas 5-HT_1A_R activation in the periaqueductal gray of high-anxious rats had the opposite effect (Ferreira and Nobre, 2014). These findings highlight how environmental conditions can contribute to individual differences in 5-HT_1A_R-mediated response differences. In line with this, single-housed mice display a stronger hypothermic effect upon 5-HT_1A_R activation by 8-OH-DPAT, which is associated with an increased depressive-like state, in comparison to their group-housed counterparts (Kalliokoski et al., 2014). However, the mechanisms underlying the inter-individual differences in serotonergic signaling and consequently in cognitive and emotional modulation are not clear yet.

A linkage disequilibrium study identified two polymorphisms (rs3808932 and rs12412496) in the human *HTR7* suggesting that it is a schizophrenia susceptibility gene (Ikeda et al., 2006). However, to the best of our knowledge, there is no evidence for the effect of 5-HT_7_R polymorphisms on serotonergic signaling or the interaction between polymorphisms of 5-HT_7_ and 5-HT_1A_Rs. Therefore, to elucidate the functional interaction between 5HT_1A_R and 5-HT_7_R, it is of high importance to understand which polymorphisms influence the expression of those 5-HTRs and how these changes affect emotional and cognitive functions. This knowledge could potentially reveal the polymorphisms that modulate the endophenotypes of different affective disorders, closely linked with the function of 5-HT_1A_R and 5-HT_7_R, such as anxiety and depression.

## Neurochemical Effects in the Hippocampus

In contrast to the above electrophysiological results, *in vivo* microdialysis in awake rats showed that the local blockade of 5-HT_1A_R increased extracellular acetylcholine (ACh) levels (Madjid et al., 2006; Hirst et al., 2008; Kehr et al., 2010) but failed to show changes in hippocampal glutamate release in the ventral hippocampus and the prefrontal cortex (Kehr et al., 2010). The result with ACh is consistent with the pro-cognitive effect of (postsynaptic) 5-HT_1A_R blockade in PA (Madjid et al., 2006). However, the expected glutamate increase may not be detectable because of the limited capacity of microdialysis to detect small transmitter changes restricted to the synaptic cleft. More sensitive techniques are required such as enzyme-based microelectrode amperometry, which is selective for the detection of extracellular glutamate with (1) spatial resolution in the μm level, (2) sub-second temporal resolution and (3) sensitivity in the μm range of glutamate (Day et al., 2006; Konradsson-Geuken et al., 2009; Mishra et al., 2015). This novel technology is suited to provide evidence for the expected enhancement of glutamatergic transmission in the hippocampus by both 5-HT_1A_R inhibition and 5-HT_7_R activation.

**FIGURE 1:**
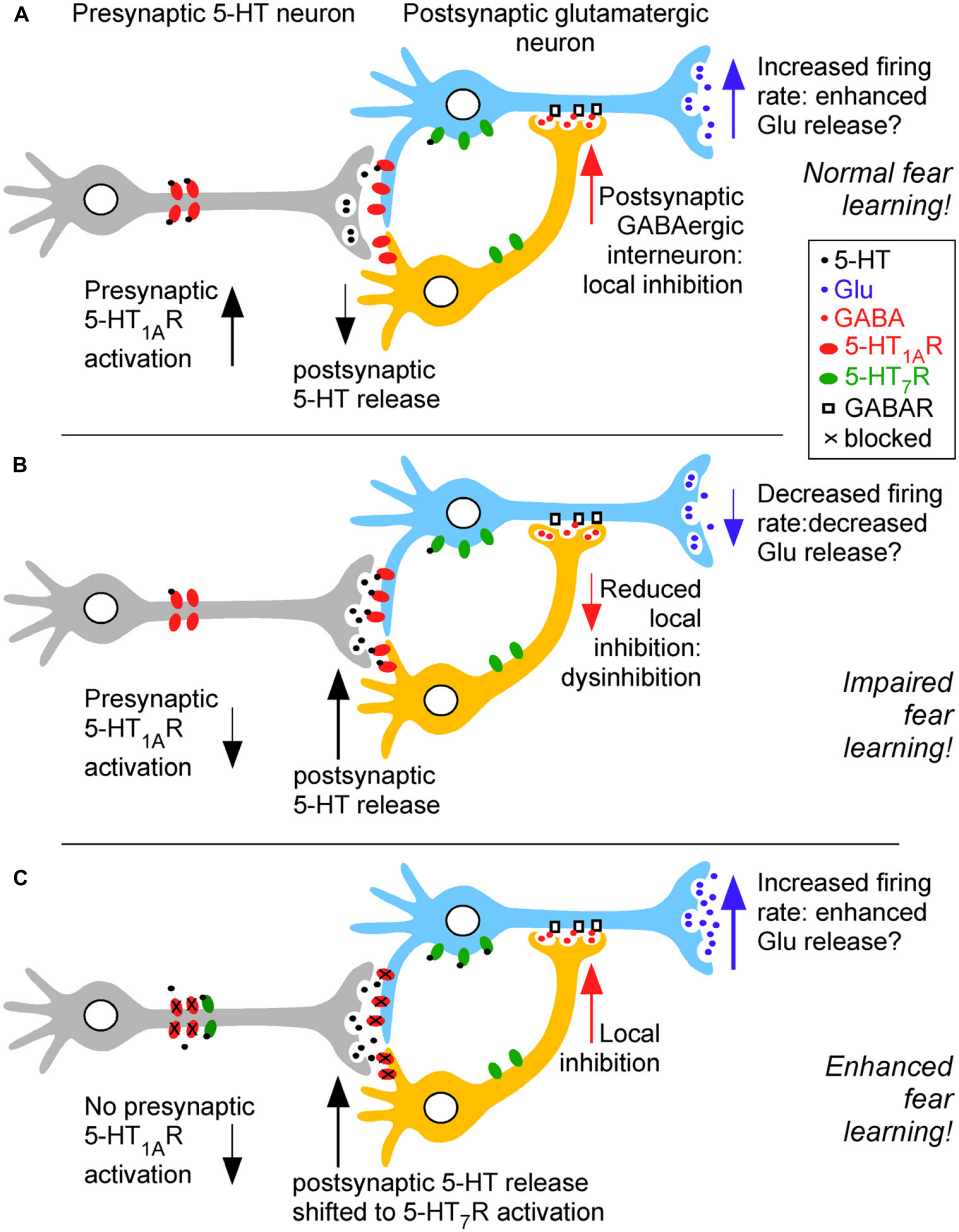
**Simplified overview of 5-HT_1A_R- and 5-HT_7_R-mediated modulation of fear learning in pre- and postsynaptic neurons under conditions of high (A) and low presynaptic 5-HT_1A_R activation (B), resulting in low and high postsynaptic 5-HT release, respectively.** This in turn causes increased and decreased acetylcholine (ACh) release in the hippocampus (and also the medial septum). A similar effect on hippocampal glutamate (Glu) levels is hypothesized (as shown in the medial septum). When high postsynaptic 5-HT levels are biased to 5-HT_7_R activation **(C)**, e.g., by 8-OH-DPAT at the postsynaptic dose of 1 mg/kg in combination with the 5-HT_1A_R antagonist NAD-299 at 0.3 mg/kg, a pro-cognitive effect in fear learning is observed. Thus, emotional learning and memory depend on intrasynaptic 5-HT levels, receptor availability and occupancy, genetic and epigenetic factors for 5-HTR regulation and its short- and long-term mechanisms underlying altered synaptic transmission via ACh and glutamate (Glu) release. Under conditions of higher (postsynaptic) 5-HT release, the cognitive consequences depend on the availability and occupancy of 5-HT_1A_R and 5-HT_7_Rs with so far unknown conditions that bias toward impaired **(B)** or facilitated fear memory **(C)**. The specific functions of GABAergic interneurons in 5-HT_1A_R and 5-HT_7_R-mediated fear memory modulation are currently not understood.

It is clear that the impairing effects of low dose NMDA receptor antagonists (e.g., MK-801) and cholinergic antagonist (e.g., scopolamine) can be prevented by serotonergic manipulations (Ögren et al., 2008). Thus, these two pharmacological models of cognitive impairment relevant for Alzheimer’s disease are both alleviated by 5-HT_1A_R inhibition demonstrating a role for both enhanced glutamatergic and cholinergic transmission for improved cognitive function (e.g., Schechter et al., 2005; Madjid et al., 2006). An overview of these modulatory effects is provided in **Figure 1**.

## Conclusion and Future Perspectives

During the last three decades many studies have indicated important regulatory functions of 5-HT signaling for emotional and cognitive functions. However, the complexity of the serotonergic signaling due to the existence of at least 14 pre- and postsynaptic 5-HTRs subtypes with multiple transduction mechanisms makes it exceedingly difficult to assign unambiguously the physiological and behavioral role of a single 5-HTR subtype. However, the use of specific ligands in combination with systemic and intrahippocampal administration, receptor autoradiography and *in vivo* neurochemical measurements are powerful tools in identifying the action of specific ligands in local networks of the brain including subareas of the hippocampus. This approach, combined with *in vivo* electrophysiology and genetic tools, can also better define the functional role of 5-HT in the neuronal circuitry underlying cognitive function.

Overall a number of open questions need to be answered to further improve our understanding of the role of serotonergic signaling via the different 5-HTRs in health and disease:

(1) How do 5-HTRs modulate hippocampal and cortical glutamatergic transmission with a focus on activation and inhibition of 5-HT_1A_Rs and 5-HT_7_Rs? This needs to be determined with newly developed amperometry methods in *in vivo* recordings.
(2) What are the roles of 5-HT_1A_Rs and 5-HT_7_Rs in defined hippocampal subregions for emotional and cognitive functions? This requires the development of new ligands with low lipophilicity for local actions tested *in vivo*. Alternatively, is should be possible to shut down the second messenger coupling of neurons selectively expressing 5-HT_1A_Rs and 5-HT_7_Rs by Designer Receptors Exclusively Activated by Designer Drugs (DREADD) technology.
(3) What are the roles of 5-HT_1A_Rs and 5-HT_7_Rs in different memory phases? As indicated there are considerable inconsistencies about the role of 5-HT_1A_Rs and 5-HT_7_Rs in the consolidation process. In addition, extinction and reconsolidation are so far poorly explored.
(4) The regulation of 5-HTR expression has so far focused on the 5-HT_1A_R. This needs to be extended to other 5-HTRs including the 5-HT_7_R. Besides the use of radio-ligands in imaging studies, the subcellular immunohistochemical analyses of 5-HTR protein levels requires the development of specific antibodies.
(5) Finally, despite the evidence of the beneficial effects of 5-HT_1A_R antagonists in preclinical models, the therapeutic potential to facilitate cholingergic and/or glutamatergic neurotransmission for improved cognitive function in human neuropathology (e.g., Alzheimer’s disease) or in aging is so far not explored.

## Conflict of Interest Statement

The authors declare that the research was conducted in the absence of any commercial or financial relationships that could be construed as a potential conflict of interest.

## Abbreviations

5-HT: 5-hydroxytryptamine
5-HTR: 5-HT receptor
cAMP: cyclic AMP
CNS: central nervous system
Epac: exchange proteins directly activated by cAMP
ERK: extracellular signal-related kinase
FC: fear conditioning
HR: heart rate
MAPK: mitogen-activated protein kinase
PA: passive avoidance
PKA: protein kinase A
SSRI: selective serotonin reuptake inhibitor

## References

[B1] Ahlenius S. Larsson K. Wijkström A. (1991). Behavioral and biochemical effects of the 5-HT1A receptor agonists flesinoxan and 8-OH-DPAT in the rat. *Eur. J. Pharmacol.* 200 259–266. 10.1016/0014-2999(91)90580-J 1838333

[B2] Allen A. R. Singh A. Zhuang Z. P. Kung M. P. Kung H. F. Lucki I. (1997). The 5-HT1A receptor antagonist p-MPPI blocks responses mediated by postsynaptic and presynaptic 5-HT1A receptors. *Pharmacol. Biochem. Behav.* 57 301–307. 10.1016/S0091-3057(96)00339-5 9164586

[B3] Almada R. C. Borelli K. G. Albrechet-Souza L. Brandão M. L. (2009). Serotonergic mechanisms of the median raphe nucleus-dorsal hippocampus in conditioned fear: output circuit involves the prefrontal cortex and amygdale. *Behav. Brain Res.* 203 279–287. 10.1016/j.bbr.2009.05.017 19464321

[B4] Artigas F. (2015). Developments in the field of antidepressants, where do we go now? *Eur. Neuropsychopharmacol.* 25 657–670. 10.1016/j.euroneuro.2013.04.013 23706576

[B5] Artigas F. Celada P. Laruelle M. Adell A. (2001). How does pindolol improve antidepressant action? *Trends Pharmacol. Sci.* 22 224–228. 10.1016/S0165-6147(00)01682-5 11339972

[B6] Arvidsson L. E. Hacksell U. Nilsson J. L. Hjorth S. Carlsson A. Lindberg P. (1981). 8-Hydroxy-2-(di-n-propylamino)tetralin, a new centrally acting 5-hydroxytryptamine receptor agonist. *J. Med. Chem.* 24 9219–9223. 10.1021/jm00140a002 6460101

[B7] Assié M. B. Lomenech H. Ravailhe V. Faucillon V. Newman-Tancredi A. (2006). Rapid desensitization of somatodendritic 5-HT1A receptors by chronic administration of the high-efficacy 5-HT1A agonist, F13714: a microdialysis study in the rat. *Br. J. Pharmacol.* 149 170–178. 10.1038/sj.bjp.0706859 16921393 PMC2013794

[B8] Baarendse P. J. J. van Grootheest G. Jansen R. F. Pieneman A. W. Ögren S. O. Verhage M. (2008). Differential involvement of the dorsal hippocampus in passive avoidance in C57BL/6J and DBA/2J mice. *Hippocampus* 18 11–19. 10.1002/hipo.20356 17696168

[B9] Bard J. A. Zgombick J. Adham N. Vaysse P. Branchek T. A. Weinshank R. L. (1993). Cloning of a novel human serotonin receptor (5-HT7) positively linked to adenylate cyclase. *J. Biol. Chem.* 268 23422–23426. 8226867

[B10] Barnes N. M. Sharp T. (1999). A review of central 5-HT receptors and their function. *Neuropharmacology* 38 1083–1152. 10.1016/S0028-3908(99)00010-6 10462127

[B11] Bauer E. P. (2014). Serotonin in fear conditioning processes. *Behav. Brain Res.* 15 68–77. 10.1016/j.bbr.2014.07.028 25078294

[B12] Becker O. M. Dhanoa D. S. Marantz Y. Chen D. Shacham S. Cheruku S. (2006). An integrated in silico 3D model-driven discovery of a novel, potent, and selective amidosulfonamide 5-HT1A agonist (PRX-00023) for the treatment of anxiety and depression. *J. Med. Chem.* 49 3116–3135. 10.1021/jm0508641 16722631

[B13] Béïque J. C. Imad M. Mladenovic L. Gingrich J. A. Andrade R. (2007). Mechanism of the 5-hydroxytryptamine 2A receptor-mediated facilitation of synaptic activity in prefrontal cortex. *Proc. Natl. Acad. Sci. U.S.A.* 104 9870–9875. 10.1073/pnas.0700436104 17535909 PMC1887564

[B14] Berger M. Gray J. A. Roth B. L. (2009). The expanded biology of serotonin. *Annu. Rev. Med.* 60 355–366. 10.1146/annurev.med.60.042307.110802 19630576 PMC5864293

[B15] Bertrand F. Lehmann O. Galani R. Lazarus C. Jeltsch H. Cassel J. C. (2001). Effects of MDL 73005 on water-maze performances and locomotor activity in scopolamine-treated rats. *Pharmacol. Biochem. Behav.* 68 647–660. 10.1016/S0091-3057(01)00448-8 11526961

[B16] Berumen L. C. Rodríguez A. Miledi R. García-Alcocer G. (2012). Serotonin receptors in hippocampus. *Sci. World J.* 2012:823493 10.1100/2012/823493 PMC3353568 22629209

[B17] Bickmeyer U. Heine M. Manzke T. Richter D. W. (2002). Differential modulation of Ih by 5-HT receptors in mouse CA1 hippocampal neurons. *Eur. J. Neurosci.* 16 209–218. 10.1046/j.1460-9568.2002.02072.x 12169103

[B18] Blier P. Ward N. M. (2003). Is there a role for 5-HT1A agonists in the treatment of depression? *Biol. Psychiatry* 53 193–203. 10.1016/S0006-3223(02)01643-8 12559651

[B19] Bockaert J. Claeysen S. Bécamel C. Dumuis A. Marin P. (2006). Neuronal 5-HT metabotropic receptors: fine-tuning of their structure, signaling, and roles in synaptic modulation. *Cell Tiss. Res.* 326 553–572. 10.1007/s00441-006-0286-1 16896947

[B20] Bonaventure P. Nepomuceno D. Hein L. Sutcliffe J. G. Lovenberg T. Hedlund P. B. (2004). Radioligand binding analysis of knockout mice reveals 5-hydroxytryptamine7 receptor distribution and uncovers 8-hydroxy-2-(di-n-propylamino)tetralin interaction with 2 adrenergic receptors. *Neuroscience* 124 901–911. 10.1016/j.neuroscience.2004.01.014 15026130

[B21] Borelli K. G. Gárgaro A. C. dos Santos J. M. Brandão M. L. (2005). Effects of inactivation of serotonergic neurons of the median raphe nucleus on learning and performance of contextual fear conditioning. *Neurosci. Lett.* 387 105–110. 10.1016/j.neulet.2005.07.031 16085359

[B22] Bosker F. J. Folgering J. H. Gladkevich A. V. Schmidt A. van der Hart M. C. Sprouse J. (2009). Antagonism of 5-HT1A receptors uncovers an excitatory effect of SSRIs on 5-HT neuronal activity, an action probably mediated by 5-HT7 receptors. *J. Neurochem.* 108 1126–1135. 10.1111/j.1471-4159.2008.05850.x 19166502

[B23] Bradley P. B. Engle G. Feniuk W. Fozard J. R. Humphrey P. P. A. Middlemis D. N. (1986). Proposals for the classification and nomenclature of functional receptors of 5-hydroxytryptamine. *Neuropharmacology* 25 563–576. 10.1016/0028-3908(86)90207-8 2875415

[B24] Brenchat A. Romero L. García M. Pujol M. Burgueño J. Torrens A. (2009). 5-HT7 receptor activation inhibits mechanical hypersensitivity secondary to capsaicin sensitization in mice. *Pain* 141 239–247. 10.1016/j.pain.2008.11.009 19118950

[B25] Cahill L. McGaugh J. L. (1998). Mechanisms of emotional arousal and lasting declarative memory. *Trends Neurosci.* 21 294–299. 10.1016/S0166-2236(97)01214-9 9683321

[B26] Carli M. Balducci C. Millan M. J. Bonalumi P. Samanin R. (1999). S 15535, a benzodioxopiperazine acting as presynaptic agonist and postsynaptic 5-HT1A receptor antagonist, prevents the impairment of spatial learning caused by intrahippocampal scopolamine. *Br. J. Pharmacol.* 128 1207–1214. 10.1038/sj.bjp.0701632 10578133 PMC1571756

[B27] Carli M. Lazarova M. Tatarczynska E. Samanin R. (1992). Stimulation of 5-HT1A receptors in the dorsal hippocampus impairs acquisition and performance of a spatial task in a water maze. *Brain Res.* 595 50–56. 10.1016/0006-8993(92)91451-J 1467958

[B28] Celada P. Puig M. V. Artigas F. (2013). Serotonin modulation of cortical neurons and networks. *Front. Integr. Neurosci.* 7:25 10.3389/fnint.2013.00025 PMC3630391 23626526

[B29] Charnay Y. Léger L. (2010). Brain serotonergic circuitries. *Dialogues Clin. Neurosci.* 12 471–487. 21319493 10.31887/DCNS.2010.12.4/ycharnay PMC3181988

[B30] Choi J. Y. Shin S. Lee M. Jeon T. J. Seo Y. Kim C. H. (2014). Acute physical stress induces the alteration of the serotonin 1A receptor density in the hippocampus. *Synapse* 68 363–368. 10.1002/syn.21748 24771590

[B31] Clarke W. P. Yocca F. D. Maayani S. (1996). Lack of 5-hydroxytryptamine 1A-mediated inhibition of adenylyl cyclase in dorsal raphe of male and female rats. *J. Pharmacol. Exp. Ther.* 277 1259–1266. 8667186

[B32] Costa L. Trovato C. Musumeci S. A. Catania M. V. Ciranna L. (2012). 5-HT1A and 5-HT7 receptors differently modulate AMPA receptor-mediated hippocampal synaptic transmission. *Hippocampus* 22 790–801. 10.1002/hipo.20940 21538661

[B33] Day B. K. Pomerleau F. Burmeister J. J. Huettl P. Gerhardt G. A. (2006). Microelectrode array studies of basal and potassium-evoked release of L-glutamate in the anesthetized rat brain. *J. Neurochem.* 96 1626–1635. 10.1111/j.1471-4159.2006.03673.x 16441510

[B34] De Vivo M. Maayani S. (1986). Characterization of the 5-hydroxytryptamine1A receptor-mediated inhibition of forskolin-stimulated adenylate cyclase activity in guinea pig and rat hippocampal membranes. *J. Pharmacol. Exp. Ther.* 238 248–253. 2941565

[B35] De Vry J. Schohe-Loop R. Heine H. G. Greuel J. M. Mauler F. Schmidt B. (1998). Characterization of the aminomethylchroman derivative BAY×3702 as a highly potent 5-hydroxytryptamine1A receptor agonist. *J. Pharmacol. Exp. Ther.* 284 1082–1094. 9495870

[B36] Del Tredici A. L. Schiffer H. H. Burstein E. S. Lameh J. Mohell N. Hacksell U. (2004). Pharmacology of polymorphic variants of the human 5-HT1A receptor. *Biochem. Pharmacol.* 67 479–490. 10.1038/sj.bjp.0705576 15037200

[B37] Den Boer J. A. Bosker F. J. Slaap B. R. (2000). Serotonergic drugs in the treatment of depressive and anxiety disorders. *Hum. Psychopharmacol.* 15 315–336. 10.1002/1099-1077(200007)15:5<315::AID-HUP204>3.0.CO;2-Y 12404310

[B38] Deseure K. Koek W. Colpaert F. C. Adriaensen H. (2002). The 5-HT1A receptor agonist F 13640 attenuates mechanical allodynia in a rat model of trigeminal neuropathic pain. *Eur. J. Pharmacol.* 456 51–57. 10.1016/j.bcp.2003.09.030 12450569

[B39] Elvander-Tottie E. Eriksson T. M. Sandin J. Ögren S. O. (2009). 5-HT1A and NMDA receptors interact in the rat medial septum and modulate hippocampal-dependent spatial learning. *Hippocampus* 19 1187–1198. 10.1002/hipo.20596 19309036

[B40] Eriksson T. M. Holst S. Stan T. L. Hager T. Sjögren B. Ögren S. O. (2012). 5-HT1A and 5-HT7 receptor crosstalk in the regulation of emotional memory: implications for effects of selective serotonin reuptake inhibitors. *Neuropharmacology* 63 1150–1160. 10.1016/j.neuropharm.2012.06.061 22801295

[B41] Eriksson T. M. Golkar A. Ekström J. C. Svenningsson P. Ögren S. O. (2008). 5-HT7 receptor stimulation by 8-OH-DPAT counteracts the impairing effect of 5-HT1A receptor stimulation on contextual learning in mice. *Eur. J. Pharmacol.* 596 107–110. 10.1016/j.ejphar.2008.08.026 18789922

[B42] Errico M. Crozier R. A. Plummer M. R. Cowen D. S. (2001). 5-HT7 receptors activate the mitogen activated protein kinase extracellular signal related kinase in cultured rat hippocampal neurons. *Neuroscience* 102 361–367. 10.1016/S0306-4522(00)00460-7 11166122

[B43] Ferreira R. Nobre M. J. (2014). Conditioned fear in low- and high-anxious rats is differentially regulated by cortical subcortical and midbrain 5-HT receptors. *Neuroscience* 268 159–168. 10.1016/j.neuroscience.2014.03.005 24657773

[B44] Filip M. Przegaliński E. (1996). Effects of MP-3022 on the 8-OH-DPAT-induced discriminative stimulus in rats. *Pol. J. Pharmacol.* 48 397–402. 9112679

[B45] Fletcher A. Forster E. A. Bill D. J. Brown G. Cliffe I. A. Hartley J. E. (1996). Electrophysiological, biochemical, neurohormonal and behavioural studies with WAY-100635, a potent, selective and silent 5 HT1A receptor antagonist. *Behav. Brain Res.* 73 337–353. 10.1016/0166-4328(96)00118-0 8788530

[B46] Forbes I. T. Dabbs S. Duckworth D. M. Jennings A. J. King F. D. Lovell P. J. (1998). (R)-3,N-dimethyl-N-[1-methyl-3-(4-methyl-piperidin-1-yl) propyl]benzenesulfonamide: the first selective 5-HT7 receptor antagonist. *J. Med. Chem.* 41 655–657. 10.1021/jm970519e 9513592

[B47] Gozlan H. El Mestikawy S. Pichat L. Glowinski J. Hamon M. (1983). Identification of presynaptic serotonin autoreceptors using a new ligand: 3H-PAT. *Nature* 305 140–142. 10.1038/305140a0 6225026

[B48] Grasby P. M. Sharp T. Allen T. Grahame-Smith D. G. (1992). The putative 5-HT1A antagonist BMY 7378 blocks 8-OH-DPAT-induced changes in local cerebral glucose utilization in the conscious rat. *Neuropharmacology* 31 547–551. 10.1016/0028-3908(92)90186-S 1407394

[B49] Greuel J. M. Glaser T. (1992). The putative 5-HT1A receptor antagonists NAN-190 and BMY 7378 are partial agonists in the rat dorsal raphe nucleus in vitro. *Eur. J. Pharmacol.* 211 211–219. 10.1016/0014-2999(92)90531-8 1535319

[B50] Griebel G. Misslin R. Pawlowski M. Guardiola Lemaitre B. Guillaumet G. Bizot- Espiard J. (1992). Anxiolytic-like effects of a selective 5-HT1A agonist, S20244, and its enantiomers in mice. *NeuroReport* 3 84–86. 10.1097/00001756-199201000-00022 1351756

[B51] Groenink L. Joordens R. J. Hijzen T. H. Dirks A. Olivier B. (2000). Infusion of flesinoxan into the amygdala blocks the fear-potentiated startle. *NeuroReport* 11 2285–2288. 10.1097/00001756-200007140-00043 10923686

[B52] Hadrava V. Blier P. Dennis T. Ortemann C. de Montigny C. (1995). Characterization of 5-hydroxytryptamine 1A properties of flesinoxan: in vivo electrophysiology and hypothermia study. *Neuropharmacology* 34 1311–1326. 10.1016/0028-3908(95)00098-Q 8570029

[B53] Hager T. Jansen R. F. Pieneman A. W. Manivannan S. N. Golani I. van der Sluis S. (2014). Display of individuality in avoidance behavior and risk assessment of inbred mice. *Front. Behav. Neurosci.* 8:314 10.3389/fnbeh.2014.00314 PMC4165351 25278853

[B54] Hajós-Korcsok E. McQuade R. Sharp T. (1999). Influence of 5-HT1A receptors on central noradrenergic activity: microdialysis studies using (±)-MDL 73005EF and its enantiomers. *Neuropharmacology* 38 299–306. 10.1016/S0028-3908(98)00175-0 10218872

[B55] Hannon J. Hoyer D. (2008). Molecular biology of 5-HT receptors. *Behav. Brain Res.* 195 198–213. 10.1016/j.bbr.2008.03.020 18571247

[B56] Heisler L. K. Chu H. M. Brennan T. J. Danao J. A. Bajwa P. Parsons L. H. (1998). Elevated anxiety and antidepressant-like responses in serotonin 5-HT1A receptor mutant mice. *Proc. Natl. Acad. Sci. U.S.A.* 95 15049–15054. 10.1073/pnas.95.25.15049 9844013 PMC24573

[B57] Hervas I. Artigas F. (1998). Effect of fluoxetine on extracellular 5-hydroxytryptamine in rat brain. Role of 5-HT autoreceptors. *Eur. J. Pharmacol.* 358 9–18. 10.1016/S0014-2999(98)00579-2 9809863

[B58] Heusler P. Palmier C. Tardif S. Bernois S. Colpaert F. C. Cussac D. (2010). [3H]-F13640, a novel, selective and high-efficacy serotonin 5-HT1A receptor agonist radioligand. *Naunyn Schmiedebergs Arch. Pharmacol.* 382 321–330. 10.1007/s00210-010-0551-4 20799027

[B59] Hirst W. D. Andree T. H. Aschmies S. Childers W. E. Comery T. A. Dawson L. A. (2008). Correlating efficacy in rodent cognition models with in vivo 5-hydroxytryptamine1a receptor occupancy by a novel antagonist, (R)-N-(2-methyl-(4-indolyl-1-piperazinyl)ethyl)-N-(2-pyridinyl)-cyclohexane carboxamide (WAY-101405). *J. Pharmacol. Exp. Ther.* 325 134–145. 10.1124/jpet.107.133082 18182558

[B60] Hjorth S. Carlsson A. (1982). Buspirone: effects on central monoaminergic transmission -possible relevance to animal experimental and clinical findings. *Eur. J. Pharmacol.* 83 299–303. 10.1016/0014-2999(82)90265-5 6129148

[B61] Holz G. G. Kang G. Harbeck M. Roe M. W. Chepurny O. G. (2006). Cell physiology of cAMP sensor Epac. *J. Physiol.* 577 5–15. 10.1113/jphysiol.2006.119644 16973695 PMC2000694

[B62] Homberg J. R. (2012). Serotonergic modulation of conditioned fear. *Scientifica* 2012 1–16. 10.6064/2012/821549 PMC3820492 24278743

[B63] Horisawa T. Ishibashi T. Nishikawa H. Enomoto T. Toma S. Ishiyama T. (2011). The effects of selective antagonists of serotonin 5-HT7 and 5-HT1A receptors on MK-801-induced impairment of learning and memory in the passive avoidance and Morris water maze tests in rats: mechanistic implications for the beneficial effects of the novel atypical antipsychotic lurasidone. *Behav. Brain Res.* 220 83–90. 10.1016/j.bbr.2011.01.034 21277905

[B64] Hoyer D. Clarke D. E. Fozard J. R. Hartig P. R. Martin G. R. Mylecharane E. J. (1994). International union of pharmacology classification of receptors for 5-hydroxytryptamine (serotonin). *Pharmacol. Rev.* 46 157–203. 7938165

[B65] Hoyer D. Hannon J. P. Martin G. R. (2002). Molecular, pharmacological and functional diversity of 5-HT receptors. *Pharmacol. Biochem. Behav.* 71 533–554. 10.1016/S0091-3057(01)00746-8 11888546

[B66] Hoyer D. Martin G. (1997). 5-HT receptor classification and nomenclature: towards a harmonization with the human genome. *Neuropharmacology* 36 419–428. 10.1016/S0028-3908(97)00036-1 9225265

[B67] Hunt S. P. Lovick T. A. (1982). The distribution of serotonin, met-enkephalin and beta-lipotropin-like immunoreactivity in neuronal perikarya of the cat brainstem. *Neurosci. Lett.* 30 139–145. 10.1016/0304-3940(82)90286-5 6180357

[B68] Ikeda M. Iwata N. Kitajima T. Suzuki T. Yamanouchi Y. Kinoshita Y. (2006). Positive association of the serotonin 5-HT7 receptor gene with schizophrenia in a Japanese population. *Neuropsychopharmacology* 31 866–871. 10.1038/sj.npp.1300901 16192982

[B69] Ishimura K. Takeuchi Y. Fujiwara K. Tominaga M. Yoshioka H. Sawada T. (1988). Quantitative analysis of the distribution of serotonin-immunoreactive cell bodies in the mouse brain. *Neurosci. Lett.* 91 265–270. 10.1016/0304-3940(88)90691-X 3185964

[B70] Jackson D. M. Bengtsson A. Johansson C. Cortizo L. Ross S. B. (1994). Development of tolerance to 8-OH-DPAT induced blockade of acquisition of a passive avoidance response. *Neuropharmacology* 33 1003–1009. 10.1016/0028-3908(94)90159-7 7845547

[B71] Johansson L. Sohn D. Thorberg S. O. Jackson D. M. Kelder D. Larsson L. G. (1997). The pharmacological characterization of a novel selective 5-hydroxytryptamine1A receptor antagonist, NAD-299. *J. Pharmacol. Exp. Ther.* 283 216–225. 9336327

[B72] Kalliokoski O. Teilmann A. C. Jacobsen K. R. Abelson K. S. Hau J. (2014). The lonely mouse - single housing affects serotonergic signaling integrity measured by 8-OH-DPAT-induced hypothermia in male mice. *PLoS ONE* 9:e111065 10.1371/journal.pone.0111065 PMC4249803 25436462

[B73] Kehr J. Hu X. J. Yoshitake T. Wang F. H. Osborne P. Stenfors C. (2010). The selective 5-HT1A receptor antagonist NAD-299 increases acetylocholine release but not the extracellular glutamate levels in the frontal cortex and hippocampus of awake rat. *Eur. Neupsychopharmacol.* 20 487–500. 10.1016/j.euroneuro.2010.03.003 20413275

[B74] Kia H. K. Brisorgueil M. J. Hamon M. Calas A. Verge D. (1996). Ultrastructural localization of 5-hydroxytryptamine1A receptors in the rat brain. *J. Neurosci. Res.* 46 697–708. 10.1002/(SICI)1097-4547(19961215)46:6<697::AID-JNR7>3.0.CO;2-A 8978504

[B75] Kikuchi C. Nagaso H. Hiranuma T. Koyama M. (1999). Tetrahydrobenzindoles: selective antagonists of the 5-HT7 receptor. *J. Med. Chem.* 42 533–535. 10.1021/jm980519u 10052959

[B76] Kobe F. Guseva D. Jensen T. P. Wirth A. Renner U. Hess D. (2012). 5-HT7R/G12 signaling regulates neuronal morphology and function in an age-dependent manner. *J. Neurosci.* 32 2915–2930. 10.1523/JNEUROSCI.2765-11.2012 22378867 PMC3369253

[B77] Konradsson-Geuken A. Gash C. R. Alexander K. Pomerleau F. Huettl P. Gerhardt G. A. (2009). Second-by-second analysis of alpha 7 nicotine receptor regulation of glutamate release in the prefrontal cortex of awake rats. *Synapse* 63 1069–1082. 10.1002/syn.20693 19637277 PMC2759414

[B78] Kung H. F. Kung M. P. Clarke W. Maayani S. Zhuang Z. P. (1994). A potential 5-HT1A receptor antagonist: p-MPPI. *Life Sci.* 55 1459–1462. 10.1016/0024-3205(94)00686-5 7968212

[B79] Kung H. F. Stevenson D. A. Zhuang Z. P. Kung M. P. Frederick D. Hurt S. D. (1996). New 5-HT1A receptor antagonist: [3H]p-MPPF. *Synapse* 23 344–346. 10.1002/(SICI)1098-2396(199608)23:4<344::AID-SYN13>3.0.CO;2-X 8855520

[B80] LeDoux J. E. (2000). Emotion circuits in the brain. *Annu. Rev. Neurosci.* 23 155–184. 10.1146/annurev.neuro.23.1.155 10845062

[B81] Leopoldo M. (2004). Serotonin7 receptors (5-HT7Rs) and their ligands. *Curr. Med. Chem.* 11 629–661. 10.2174/0929867043455828 15032609

[B82] Leopoldo M. Lacivita E. Berardi F. Perrone R. Hedlund P. B. (2011). Serotonin 5-HT7 receptor agents: structure-activity relationships and potential therapeutic applications in central nervous system disorders. *Pharmacol. Ther.* 129 120–148. 10.1016/j.pharmthera.2010.08.013 20923682 PMC3031120

[B83] Leopoldo M. Lacivita E. De Giorgio P. Fracasso C. Guzzetti S. Caccia S. (2008). Structural modifications of N-(1,2,3,4-tetrahydronaphthalen-1-yl)-4-aryl-1-piperazinehexanamides: influence on lipophilicity and 5-HT7 receptor activity. *Part III J. Med. Chem.* 51 5813–5822. 10.1021/jm800615e 18800769

[B84] Li X. Inoue T. Abekawa T. Weng S. Nakagawa S. Izumi T. (2006). 5-HT1A receptor agonist affects fear conditioning through stimulations of the postsynaptic 5-HT1A receptors in the hippocampus and amygdala. *Eur. J. Pharmacol.* 532 74–80. 10.1016/j.ejphar.2005.12.008 16460727

[B85] Lin S. L. Johnson-Farley N. N. Lubinsky D. R. Cowen D. S. (2003). Coupling of neuronal 5-HT7 receptors to activation of extracellular-regulated kinase through a protein kinase A-independent pathway that can utilize Epac. *J. Neurochem.* 87 1076–1085. 10.1046/j.1471-4159.2003.02076.x 14622088

[B86] Lovell P. J. Bromidge S. M. Dabbs S. Duckworth D. M. Forbes I. T. Jennings A. J. (2000). A novel, potent, and selective 5-HT7 antagonist: (R)-3-(2-(2-(4-methylpiperidin-1-yl)ethyl)pyrrolidine-1-sulfonyl)phenol (SB-269970). *J. Med. Chem.* 10 342–345. 10.1021/jm990412m 10669560

[B87] Lovenberg T. W. Baron B. M. de Lecea L. Miller J. D. Prosser R. A. Rea M. A. (1993). A novel adenylyl cyclase-activating serotonin receptor (5-HT7) implicated in the regulation of mammalian circadian rhythms. *Neuron* 11 449–458. 10.1016/0896-6273(93)90149-L 8398139

[B88] Lucki I. J. (1991). Behavioral studies of serotonin receptor agonists as antidepressant drugs. *Clin. Psychiatry* 52 24–31. 1684363

[B89] Lüttgen M. Ögren S. O. Meister B. (2005a). 5-HT1A receptor mRNA and immunoreactivity in the rat medial septum/diagonal band of Broca - relationships to GABAergic and cholinergic neurons. *J. Chem. Neuroanat.* 29 93–111. 10.1016/j.jchemneu.2004.09.001 15652697

[B90] Lüttgen M. Elvander E. Madjid N. Ögren S. O. (2005b). Analysis of the role of 5-HT1A receptors in spatial and aversive learning in the rat. *Neuropharmacology* 48 830–852. 10.1016/j.neuropharm.2005.01.007 15829255

[B91] Madjid N. Tottie E. E. Lüttgen M. Meister B. Sandin J. Kuzmin A. (2006). 5-Hydroxytryptamine 1A receptor blockade facilitates aversive learning in mice: interactions with cholinergic and glutamatergic mechanisms. *J. Pharmacol. Exp. Ther.* 316 581–591. 10.1124/jpet.105.092262 16223872

[B92] Mahé C. Loetscher E. Feuerbach D. Müller W. Seiler M. P. Schoeffter P. (2004). Differential inverse agonist efficacies of SB-258719, SB-258741 and SB-269970 at human recombinant serotonin 5-HT7 receptors. *Eur. J. Pharmacol.* 495 97–102. 10.1016/j.ejphar.2004.05.033 15249157

[B93] Martin-Cora F. J. Pazos A. (2004). Autoradiographic distribution of 5-HT7 receptors in the human brain using [3H]mesulergine: comparison to other mammalian species. *Br. J. Pharmacol.* 141 92–104. 10.1038/sj.bjp.0705576 14656806 PMC1574165

[B94] Matsuda T. Yoshikawa T. Suzuki M. Asano S. Somboonthum P. Takuma K. (1995). Novel benzodioxan derivative, 5-(3-[((2S)-1,4-benzodioxan-2-ylmethyl)amino]propoxy)-1,3-benzodioxole HCl (MKC-242), with a highly potent and selective agonist activity at rat central serotonin1A receptors. *Jpn. J. Pharmacol.* 69 357–366. 10.1254/jjp.69.357 8786639

[B95] Maurel J. L. Autin J. M. Funes P. Newman-Tancredi A. Colpaert F. Vacher B. (2007). High-efficacy 5-HT1A agonists for antidepressant treatment: a renewed opportunity. *J. Med. Chem.* 50 5024–5033. 10.1021/jm070714l 17803293

[B96] Mendelson S. D. Quartermain D. Francisco T. Shemer A. (1993). 5-HT_1A_ receptor agonists induce anterograde amnesia in mice through a postsynaptic mechanism. *Eur. J. Pharmacol.* 236 177–182. 10.1016/0014-2999(93)90587-8 8319749

[B97] Meneses A. (2004). Effects of the 5-HT_7_ receptor antagonists SB-269970 and DR 4004 in autoshaping Pavlovian/instrumental learning task. *Behav. Brain Res.* 155 275–282. 10.1016/j.bbr.2004.04.026 15364487

[B98] Meneses A. Perez-García G. Liy-Salmeron G. Ponce-López T. Lacivita E. Leopoldo M. (2015). 5-HT7 receptor activation: procognitive and antiamnesic effects. *Psychopharmacology* 232 595–603. 10.1007/s00213-014-3693-0 25074446

[B99] Millan M. J. Agid Y. Brüne M. Bullmore E. T. Carter C. S. Clayton N. S. (2012). Cognitive dysfunction in psychiatric disorders: characteristics, causes and the quest for improved therapy. *Nat. Rev. Drug. Discov.* 11 141–168. 10.1038/nrd3628 22293568

[B100] Millan M. J. Rivet J. M. Canton H. Lejeune F. Gobert A. Widdowson P. (1993). S 15535, a highly selective benzodioxopiperazine 5-HT1A receptor ligand which acts as an agonist and an antagonist at presynaptic and postsynaptic sites respectively. *Eur. J. Pharmacol.* 230 99–102. 10.1016/0014-2999(93)90416-F 8381359

[B101] Minabe Y. Schechter L. Hashimoto K. Shirayama Y. Ashby C. R. Jr. (2003). Acute and chronic administration of the selective 5-HT1A receptor antagonist WAY-405 significantly alters the activity of midbrain dopamine neurons in rats: an in vivo electrophysiological study. *Synapse* 50 181–190. 10.1002/syn.10255 14515335

[B102] Misane I. Johansson C. Ögren S. O. (1998). Analysis of the 5-HT1A receptor involvement in passive avoidance in the rat. *Br. J. Pharmacol.* 125 499–509. 10.1038/sj.bjp.0702098 9806333 PMC1565647

[B103] Misane I. Kruis A. Pieneman A. W. Ögren S. O. Stiedl O. (2013). GABAA receptor activation in the CA1 area of the dorsal hippocampus impairs consolidation of conditioned contextual fear in C57BL/6J mice. *Behav. Brain Res.* 238 160–169. 10.1016/j.bbr.2012.10.027 23098796

[B104] Misane I. Ögren S. O. (2000). Multiple 5-HT receptors in passive avoidance: comparative studies of p-chloroamphetamine and 8-OH-DPAT. *Neuropsychopharmacology* 22 168–190. 10.1016/S0893-133X(99)00109-8 10649830

[B105] Mishra D. Harrison N. R. Gonzales C. B. Schilström B. Konradsson-Geuken Å. (2015). Effects of age and acute ethanol on glutamatergic neurotransmission in the medial prefrontal cortex of freely moving rats using enzyme-based microelectrode amperometry. *PLoS ONE* 10:e0125567 10.1371/journal.pone.0125567 PMC4416039 25927237

[B106] Monti J. M. Leopoldo M. Jantos H. (2008). The serotonin 5-HT7 receptor agonist LP-44 microinjected into the dorsal raphe nucleus suppresses REM sleep in the rat. *Behav. Brain Res.* 191 184–189. 10.1016/j.bbr.2008.03.025 18466985

[B107] Moreau J. L. Griebel G. Jenck F. Martin J. R. Widmer U. Haefely W. E. (1992). Behavioral profile of the 5-HT1A receptor antagonist (S)-UH-301 in rodents and monkeys. *Brain Res. Bull.* 29 901–904. 10.1016/0361-9230(92)90163-R 1473022

[B108] Naumenko V. S. Popova N. K. Lacivita E. Leopoldo M. Ponimaskin E. G. (2014). Interplay between serotonin 5-HT1A and 5-HT7 receptors in depressive disorders. *CNS Neurosci. Ther.* 20 582–590. 10.1111/cns.12247 24935787 PMC6493079

[B109] Newman-Tancredi A. Martel J. C. Assié M. B. Buritova J. Lauressergues E. Cosi C. (2009). Signal transduction and functional selectivity of F15599, a preferential post-synaptic 5-HT1A receptor agonist. *Br. J. Pharmacol.* 156 338–353. 10.1111/j.1476-5381.2008.00001.x 19154445 PMC2697830

[B110] Nichols D. E. Nichols C. D. (2008). Serotonin receptors. *Chem. Rev.* 108 1614–1641. 10.1021/cr078224o 18476671

[B111] Norum J. H. Hart K. Levy F. O. (2003). Ras-dependent ERK activation by the human Gs-coupled serotonin receptors 5-HT4(b) and 5-HT7(a). *J. Biol. Chem.* 278 3098–3104. 10.1074/jbc.M206237200 12446729

[B112] Ögren S. O. Eriksson T. M. Elvander-Tottie E. D’Addario C. Ekström J. C. Svenningsson P. (2008). The role of 5-HT1A receptors in learning and memory. *Behav. Brain Res.* 195 54–77. 10.1016/j.bbr.2008.02.023 18394726

[B113] Ögren S. O. Stiedl O. (2015). “Passive avoidance,” in *Encyclopedia of Psychopharmacology*, 2nd Edn, eds Stolerman I. P. Lawrence H. Price L. H. (Berlin: Springer), 1220–1228. 10.1007/978-3-642-36172-2_160

[B114] Parks C. L. Robinson P. S. Sibille E. Shenk T. Toth M. (1998). Increased anxiety of mice lacking the serotonin 1A receptor. *Proc. Natl. Acad. Sci. U.S.A.* 95 10734–10739. 10.1073/pnas.95.18.10734 9724773 PMC27964

[B115] Passchier J. van Waarde A. Pieterman R. M. Elsinga P. H. Pruim J. Hendrikse H. N. (2000). In vivo delineation of 5-HT1A receptors in human brain with [18F]MPPF. *J. Nucl. Med.* 41 1830–1835. 11079490

[B116] Perez-García G. S. Meneses A. (2005). Effects of the potential 5-HT7 receptor agonist AS 19 in an autoshaping learning task. *Behav. Brain Res.* 163 136–140. 10.1016/j.bbr.2005.04.014 15936093

[B117] Pike V. W. McCarron J. A. Lammertsma A. A. Osman S. Hume S. P. Sargent P. A. (1996). Exquisite delineation of 5-HT1A receptors in human brain with PET and [carbonyl-11C]WAY-100635. *Eur. J. Pharmacol.* 301 R5–R7. 10.1016/0014-2999(96)00079-9 8773468

[B118] Pitsikas N. Rigamonti A. E. Cella S. G. Muller E. E. (2003). The 5-HT1A receptor antagonist WAY 100635 improves rats performance in different models of amnesia evaluated by the object recognition task. *Brain Res.* 983 215–222. 10.1016/S0006-8993(03)03091-9 12914983

[B119] Pittalà V. Siracusa M. A. Salerno L. Romeo G. Modica M. N. Madjid N. (2015). Analysis of mechanisms for memory enhancement using novel and potent 5-HT1A receptor ligands. *Eur. Neuropsychopharmacol.* 10.1016/j.euroneuro.2015.04.017 [Epub ahead of print]. 25963581

[B120] Pouzet B. Didriksen M. Arnt J. (2002). Effects of the 5-HT7 receptor antagonist SB-258741 in animal models for schizophrenia. *Pharmacol. Biochem. Behav.* 71 655–665. 10.1016/S0091-3057(01)00744-4 11888557

[B121] Quartermain D. Clemente J. Shemer A. (1993). 5-HT1A agonists disrupt memory of fear conditioning in mice. *Biol. Psychiatry* 33 247–254. 10.1016/0006-3223(93)90290-T 8471677

[B122] Raghupathi R. K. Rydelek-Fitzgerald L. Teitler M. Glennon R. A. (1991). Analogues of the 5-HT1A serotonin antagonist 1-(2-methoxyphenyl)-4-[4-(2-phthalimido) butyl]piperazine with reduced alpha 1-adrenergic affinity. *J. Med. Chem.* 34 2633–2638. 10.1021/jm00112a043 1652026

[B123] Rang H. P. Ritter J. M. Flower R. J. Henderson G. (2015). *Pharmacology*, 8th Edn (London: Elsevier Churchill Livingstone).

[B124] Rasmussen K. Calligaro D. O. Czachura J. F. Dreshfield-Ahmad L. J. Evans D. C. Hemrick-Luecke S. K. (2000). The novel 5-hydroxytryptamine1A antagonist LY426965: effects on nicotine withdrawal and interactions with fluoxetine. *J. Pharmacol. Exp. Ther.* 294 688–700. 10900249

[B125] Raymond J. R. Mukhin Y. V. Gelasco A. Turner J. Collinsworth G. Gettys T. W. (2001). Multiplicity of mechanisms of serotonin receptor signal transduction. *Pharmacol. Ther.* 92 179–212. 10.1016/S0163-7258(01)00169-3 11916537

[B126] Renner U. Zeug A. Woehler A. Niebert M. Dityatev A. Dityateva G. (2012). Heterodimerization of serotonin receptors 5-HT1A and 5-HT7 differentially regulates receptor signalling and trafficking. *Cell Sci.* 15 2486–2499. 10.1242/jcs.101337 22357950

[B127] Riad M. Garcia S. Watkins K. C. Jodoin N. Doucet E. Langlois X. (2000). Somatodendritic localization of 5-HT1A and preterminal axonal localization of 5-HT1B serotonin receptors in adult rat brain. *J. Comp. Neurol.* 417 181–194. 10.1002/(SICI)1096-9861(20000207)417:2<181::AID-CNE4>3.0.CO;2-A 10660896

[B128] Roberts A. J. Krucker T. Levy C. L. Slanina K. A. Sutcliffe J. G. Hedlund P. B. (2004). Mice lacking 5-HT receptors show specific impairments in contextual learning. *Eur. J. Neurosci.* 19 1913–1922. 10.1111/j.1460-9568.2004.03288.x 15078565

[B129] Rotondo A. Nielsen D. A. Nakhai B. Hulihan-Giblin B. Bolos A. Goldman D. (1997). Agonist promoted down-regulation and functional desensitization in two naturally occurring variants of the human serotonin1A receptor. *Neuropsychopharmacology* 17 18–26. 10.1016/S0893-133X(97)00021-3 9194046

[B130] Ruat M. Traiffort E. Leurs R. Tardivel-Lacombe J. Diaz J. Arrang J. M. (1993). Molecular cloning, characterization, and localization of a high-affinity serotonin receptor (5-HT7) activating cAMP formation. *Proc. Natl. Acad. Sci. U.S.A.* 90 8547–8551. 10.1073/pnas.90.18.8547 8397408 PMC47394

[B131] Sakaue M. Ago Y. Baba A. Matsuda T. (2003). The 5-HT1A receptor agonist MKC-242 reverses isolation rearing-induced deficits of prepulse inhibition in mice. *Psychopharmacology* 170 73–79. 10.1007/s00213-003-1515-x 12768276

[B132] Sarkisyan G. Hedlund P. B. (2009). The 5-HT7 receptor is involved in allocentric spatial memory information processing. *Behav. Brain Res.* 202 26–31. 10.1016/j.bbr.2009.03.011 19447277 PMC2684518

[B133] Savitz J. Lucki I. Drevets W. C. (2009). 5-HT1A receptor function in major depressive disorder. *Prog. Neurobiol.* 88 17–31. 10.1016/j.pneurobio.2009.01.009 19428959 PMC2736801

[B134] Schechter L. E. Smith D. L. Rosenzweig-Lipson S. Sukoff S. J. Dawson L. A. Marquis K. (2005). Lecozotan (SRA-333): a selective serotonin 1A receptor antagonist that enhances the stimulated release of glutamate and acetylcholine in the hippocampus and possesses cognitive-enhancing properties. *J. Pharmacol. Exp. Ther.* 314 1274–1289. 10.1124/jpet.105.086363 15951399

[B135] Schwarz T. Beckermann B. Buehner K. Mauler F. Schuhmacher J. Seidel D. (2005). Pharmacokinetics of repinotan in healthy and brain injured animals. *Biopharm. Drug Dispos.* 26 259–268. 10.1002/bdd.458 15966026

[B136] Shimizu H. Hirose A. Tatsuno T. Nakamura M. Katsube J. (1987). Pharmacological properties of SM-3997: a new anxioselective anxiolytic candidate. *Jpn. J. Pharmacol.* 45 493–500. 10.1254/jjp.45.493 2895201

[B137] Siracusa M. A. Salerno L. Modica M. N. Pittalà V. Romeo G. Amato M. E. (2008). Synthesis of new arylpiperazinylalkylthiobenzimidazole, benzothiazole, or benzoxazole derivatives as potent and selective 5-HT1A serotonin receptor ligands. *J. Med. Chem.* 51 4529–4538. 10.1021/jm800176x 18598015

[B138] Skirzewski M. Hernandez L. Schechter L. E. Rada P. (2010). Acute lecozotan administration increases learning and memory in rats without affecting anxiety or behavioral depression. *Pharmacol. Biochem. Behav.* 95 325–330. 10.1016/j.pbb.2010.02.008 20170670

[B139] Sodickson D. L. Bean B. P. (1998). Neurotransmitter activation of inwardly rectifying potassium current in dissociated hippocampal CA3 neurons: interactions among multiple receptors. *J. Neurosci.* 18 8153–8162. 9763462 10.1523/JNEUROSCI.18-20-08153.1998 PMC6792863

[B140] Starr K. R. Price G. W. Watson J. M. Atkinson P. J. Arban R. Melotto S. (2007). SB-649915-B, a novel 5-HT1A/B autoreceptor antagonist and serotonin reuptake inhibitor, is anxiolytic and displays fast onset activity in the rat high light social interaction test. *Neuropsychopharmacology* 32 2163–2172. 10.1038/sj.npp.1301341 17356576

[B141] Stenfors C. Werner T. Ross S. B. (1998). In vivo labelling of the mouse brain 5-hydroxytryptamine1A receptor with the novel selective antagonist 3H-NAD-299. N.-S. *Arch. Pharmacol.* 357 500–507. 10.1007/PL00005199 9650801

[B142] Stiedl O. Jansen R. F. Pieneman A. W. Ögren S. O. Meyer M. (2009). Assessing aversive emotional states through the heart in mice: implications for cardiovascular dysregulation in affective disorders. *Neurosci. Biobehav. Rev.* 33 181–190. 10.1016/j.neubiorev.2008.08.015 18824021

[B143] Stiedl O. Misane I. Spiess J. Ögren S. O. (2000a). Involvement of the 5-HT1A receptors in classical fear conditioning in C57BL/6J mice. *J. Neurosci.* 20 8515–8527. 11069959 10.1523/JNEUROSCI.20-22-08515.2000 PMC6773161

[B144] Stiedl O. Birkenfeld K. Palve M. Spiess J. (2000b). Impairment of conditioned contextual fear of C57BL/6J mice by intracerebral injections of the NMDA receptor antagonist APV. *Behav. Brain Res.* 116 157–168. 10.1016/S0166-4328(00)00269-2 11080547

[B145] Swanson S. P. Catlow J. (1992). Disposition of the novel serotonin agonist, LY228729, in monkeys and rats. *Drug Metab. Dispos.* 20 102–107. 1346983

[B146] Takahashi H. Nakashima S. Ohama E. Takeda S. Ikuta F. (1986). Distribution of serotonin-containing cell bodies in the brainstem of the human fetus determined with immunohistochemistry using antiserotonin serum. *Brain Dev.* 8 355–365. 10.1016/S0387-7604(86)80055-9 3541662

[B147] Thomas D. R. Atkinson P. J. Hastie P. G. Roberts J. C. Middlemiss D. N. Price G. W. (2002). [3H]-SB-269970 radiolabels 5-HT7 receptors in rodent, pig and primate brain tissues. *Neuropharmacology* 42 74–81. 10.1016/S0028-3908(01)00151-4 11750917

[B148] Thomas D. R. Melotto S. Massagrande M. Gribble A. D. Jeffrey P. Stevens A. J. (2003). B-656104-A, a novel selective 5-HT7 receptor antagonist, modulates REM sleep in rats. *Br. J. Pharmacol.* 139 705–714. 10.1038/sj.bjp.0705290 12812993 PMC1573887

[B149] Thomson C. G. Beer M. S. Curtis N. R. Diggle H. J. Handford E. Kulagowski J. J. (2004). Thiazoles and thiopyridines: novel series of high affinity h5HT7 ligands. *Bioorg. Med. Chem. Lett.* 14 677–680. 10.1016/j.bmcl.2003.11.050 14741267

[B150] To Z. P. Bonhaus D. W. Eglen R. M. Jakeman L. B. (1995). Characterization and distribution of putative 5-HT7 receptors in guinea-pig brain. *Br. J. Pharmacol.* 115 107–116. 10.1111/j.1476-5381.1995.tb16327.x 7647964 PMC1908750

[B151] Tomita T. Yasui-Furukori N. Nakagami T. Tsuchimine S. Ishioka M. Kaneda A. (2014). The influence of 5-HTTLPR genotype on the association between the plasma concentration and therapeutic effect of paroxetine in patients with major depressive disorder. *PLoS ONE* 9:e98099 10.1371/journal.pone.0098099 PMC4032230 24858363

[B152] Toth M. (2003). 5-HT1A receptor knockout mouse as a genetic model of anxiety. *Eur. J. Pharmacol.* 463 177–174. 10.1016/S0014-2999(03)01280-9 12600709

[B153] Traber J. Davies M. A. Dompert W. U. Glaser T. Schuurman T. Seidel P. R. (1984). Brain serotonin receptors as a target for the putative anxiolytic TVX Q 7821. *Brain Res. Bull.* 12 741–744. 10.1016/0361-9230(84)90155-2 6541079

[B154] Tunnicliff G. (1991). Molecular basis of buspirone’s anxiolytic action. *Pharmacol. Toxicol.* 69 149–156. 10.1111/j.1600-0773.1991.tb01289.x 1796057

[B155] Villalobos-Molina R. Orozco-Mendez M. Lopez-Guerrero J. J. Gallardo-Ortiz I. A. (2005). WAY 405, a new silent 5-HT1A receptor antagonist with low affinity for vascular alpha1-adrenoceptors. *Auton. Autacoid. Pharmacol.* 25 185–189. 10.1111/j.1474-8673.2005.00350.x 16176451

[B156] Waterhouse R. N. (2003). Determination of lipophilicity and its use as a predictor of blood-brain barrier penetration of molecular imaging agents. *Mol. Imaging Biol.* 5 376–389. 10.1016/j.mibio.2003.09.014 14667492

[B157] Weiss S. Pin J. P. Sebben M. Kemp D. Sladeczek F. Gabrion J. (1986). Synaptogenesis of cultured striatal neurones in serum-free medium: a morphological and biochemical study. *Proc. Natl. Acad. Sci. U.S.A.* 83 2238–2242. 10.1073/pnas.83.7.2238 3008155 PMC323267

[B158] Wesolowska A. Nikiforuk A. Stachowicz K. Tatarczynska E. (2006). Effect of the selective 5-HT7 receptor antagonist SB 269970 in animal models of anxiety and depression. *Neuropharmacology* 51 578–586. 10.1016/j.neuropharm.2006.04.017 16828124

[B159] Witkin J. M. Barrett J. E. (1986). Interaction of buspirone and dopaminergic agents on punished behavior of pigeons. *Pharmacol. Biochem. Behav.* 24 751–756. 10.1016/0091-3057(86)90585-X 2871566

[B160] Youn J. Hager T. Misane I. Pieneman A. W. Jansen R. F. Ögren S. O. (2013). Central 5-HT1A receptor-mediated modulation of heart rate dynamics and its adjustment by conditioned and unconditioned fear in mice. *Br. J. Pharmacol.* 170 859–870. 10.1111/bph.12325 23902597 PMC3799599

[B161] Youn J. Misane I. Eriksson T. M. Millan M. J. Ögren S. O. Meyer M. (2009). Bidirectional modulation of classical fear conditioning in mice by 5-HT1A receptor ligands with contrasting intrinsic activities. *Neuropharmacology* 57 567–576. 10.1016/j.neuropharm.2009.07.011 19607850

